# Synthesis and Cytotoxic Activity of New Vindoline Derivatives Coupled to Natural and Synthetic Pharmacophores

**DOI:** 10.3390/molecules25041010

**Published:** 2020-02-24

**Authors:** András Keglevich, Leonetta Dányi, Alexandra Rieder, Dorottya Horváth, Áron Szigetvári, Miklós Dékány, Csaba Szántay, Ahmed Dhahir Latif, Attila Hunyadi, István Zupkó, Péter Keglevich, László Hazai

**Affiliations:** 1 Department of Organic Chemistry and Technology, Budapest University of Technology and Economics, Gellért tér 4., H-1111 Budapest, Hungary; danyi.leonetta@gmail.com (L.D.); riederszandra@gmail.com (A.R.); dorkahorvath03@gmail.com (D.H.); pkeglevich@mail.bme.hu (P.K.); hazai@mail.bme.hu (L.H.); 2 Spectroscopic Research Department, Gedeon Richter Plc., P. O. Box 27, H-1475 Budapest 10, Hungary; szigetvaria@richter.hu (Á.S.); m.dekany@richter.hu (M.D.);; 3 Institute of Pharmacognosy, Interdisciplinary Excellence Centre, University of Szeged, Eötvös u. 6., H-6720 Szeged, Hungary; latif.ahmed@pharmacognosy.hu (A.D.L.); hunyadi.a@pharm.u-szeged.hu (A.H.); 4 Department of Pharmacodynamics and Biopharmacy, Interdisciplinary Excellence Centre, University of Szeged, Eötvös u. 6., H-6720 Szeged, Hungary; zupko@pharm.u-szeged.hu; 5 Interdisciplinary Centre for Natural Products, University of Szeged, Eötvös u. 6., H-6720 Szeged, Hungary

**Keywords:** organic synthesis, anticancer drugs, *Vinca* alkaloids, vindoline, pharmacophores, IC_50_ values

## Abstract

New *Vinca* alkaloid derivatives were synthesized to improve the biological activity of the natural alkaloid vindoline. To this end, experiments were performed to link vindoline with various structural units, such as amino acids, a 1,2,3-triazole derivative, morpholine, piperazine and N-methylpiperazine. The structure of the new compounds was characterized by NMR spectroscopy and mass spectrometry (MS). Several compounds exhibited in vitro antiproliferative activity against human gynecological cancer cell lines with IC_50_ values in the low micromolar concentration range.

## 1. Introduction

**Figure 1:**
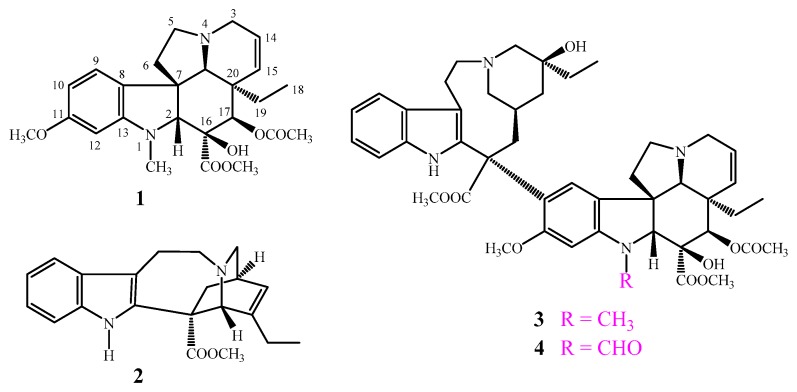
Vindoline (**1**), catharanthine (**2**), vinblastine (**3**), and vincristine (**4**) as representatives of the well-known *Vinca* alkaloid family.

The famous *Vinca* alkaloid family was isolated first in the 1950s from the leaves of *Catharanthus roseus*. Some of these natural compounds, along with their semisynthetic analogues, are still being used as chemotherapeutic agents in anticancer therapy (especially in the case of lymphomas and leukemia) [1,2,3,4,5,6,7,8,9,10,11,12,13,14,15]. Vindoline (**1**) and catharanthine (**2**) are the two subunits of the “dimeric” alkaloids vinblastine (**3**) and vincristine (**4**), which are remarkable representatives of the *Vinca* family with a significant cytotoxic activity (Figure 1). Vindoline (**1**) is present in the plant in a much larger quantity than the dimers, however, it does not have any anticancer effect.

Recently, a development program has been started aiming at the synthesis of special complex molecules by incorporating two drug pharmacophores into one single molecule so as to obtain selective anticancer drugs. The objective of this project is to make available new derivatives of *Vinca* alkaloids that could serve as alternatives to vinblastine (**3**) and vincristine (**4**), which have a favorable antitumor effect, however, also have a high molecular weight, low absorption and several unwanted effects (e.g., toxicity and multidrug-resistance). Therefore, we have attempted to test vindoline (**1**) as a potential antitumor agent by connecting it to different natural and synthetic pharmacophores.

Accordingly, our main purpose was to test the idea that not only vinblastine (**3**) and vincristine (**4**), but a “monomer” *Vinca* alkaloid unit could also have anticancer activity, when it is combined with certain structural units.

*Vinca* alkaloids are usually used in the form of sulfate salts and are administered via intravenous injection in clinical therapy since their absorption is poor from the gastrointestinal tract. Their most significant adverse effects are neurotoxicity (peripheral neuropathy) and myelosuppression. Besides their significant toxicity, multidrug-resistance (MDR) is another problem that restricts the applicability of these pharmaceutical molecules in clinical therapy. Therefore, the basic aim of our research project was to synthesize new *Vinca* alkaloid derivatives in order to increase their effectiveness and/or reduce their serious side effects.

There are several pharmaceuticals on the market, which include the pharmacophores that we wished to introduce. 1,2,3-triazole is a widely used moiety in modern drug discovery due to its advantageous structural properties (e.g., moderate dipole character, rigidity, in vivo metabolic stability and the ability to forma hydrogen bond, which increases water solubility) as a potential connecting unit. Furthermore, this particular azaheterocycle has several beneficial biological activities, such as anticancer, antifungal and antibacterial effects. There are also HIV protease and histone deacetylase inhibitors on the market, which include 1,2,3-triazole. These compounds can be easily synthesized via click chemistry, which is an increasingly used method in medicinal chemistry [16,17]. Other nitrogen-containing heterocycles, such as piperazine and morpholine could also have significant therapeutic value [18]. Piperazine analogues show diverse biological activities (e.g., antimalarial, antipsychotic, and antidepressant), too. Finally, morpholine derivatives also have outstanding pharmaceutical applications as anti-inflammatory, analgesic, neuroprotective, or antitumor agents, just to mention a few examples. The wide spectrum of biological utilities that these molecules offer made it clear that it is worthwhile to try these pharmacophores within the *Vinca* alkaloid family.

Recently, several experiments were performed to conjugate *Vinca* alkaloids with different types of amino acid esters, steroids and triphenylphosphine [19,20,21,22,23,24]. The advantage of linking with amino acids is that the given products, bound to carrier peptides (e.g., octaarginine), are able to enter directly into the cancer cells, thereby enabling more targeted therapy, and thus reducing the mentioned serious side effects. On the other hand, the steroid vector can facilitate the internalization of the drug into the cell. Finally, a triphenylphospine unit could help *Vinca* compounds to fight against multidrug resistance and promote the accumulation of the drug inside cancer cells. Moreover, it has antitumor activity on its own [25]. The products obtained showed promising half maximal inhibitory concentration (IC_50_) values [19,20,21], or were measured across the entire NCI cell panel and had promising in vitro cytotoxic activities (in terms of growth percent rates (GPR) and growth inhibition of 50% of cells (GI_50_) values) [22,23,24], according to the National Institutes of Health (NIH), US [26,27,28,29].

## 2. Results and Discussion

### 2.1. Preparation of Vindoline Derivatives Coupled to (l)- and (d)-Tryptophan Methylester

#### 2.1.1. Starting from 10-Aminovindoline (**5**) with Succinic Anhydride 

**Scheme 1:**
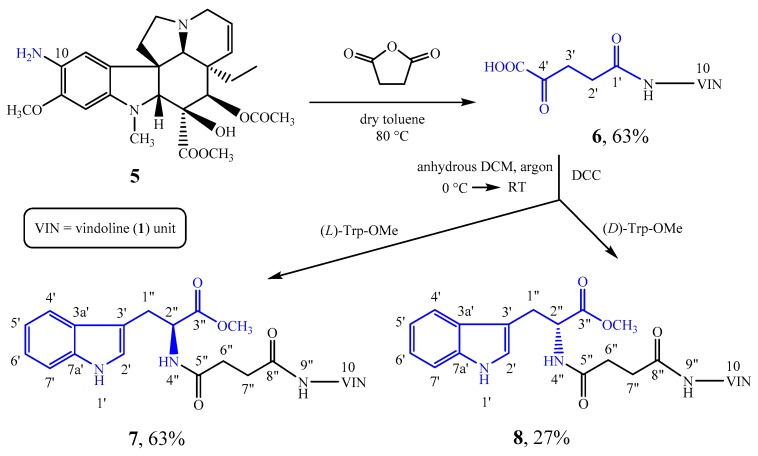
N-acylation of 10-aminovindoline (**5**) with succinic anhydride and the amidation of compound **6** with (l)- and (d)-tryptophan methyl ester.

The amino acid-conjugated vindoline derivatives (**7** and **8**) that we aimed at were achieved after the synthesis of 10-aminovindoline (**5**), known in the literature [30] (Scheme 1). Derivative **5** was N-acylated with succinic anhydride in dry toluene at 80 °C yielding compound **6**, which was then coupled with (l)- and (d)-tryptophan methylester. The amidation of compound **6** with the mentioned amino acid esters was performed in the presence of *N*,*N*’-dicyclohexylcarbodiimide (DCC) in dichloromethane (DCM) initially at 0 °C, and was then continued at room temperature. The expected products (**7** and **8**) were obtained in yields of 63% and 27%, respectively.

#### 2.1.2. Starting from 17-Desacetylvindoline (9) with Succinic Anhydride 

**Scheme 2:**
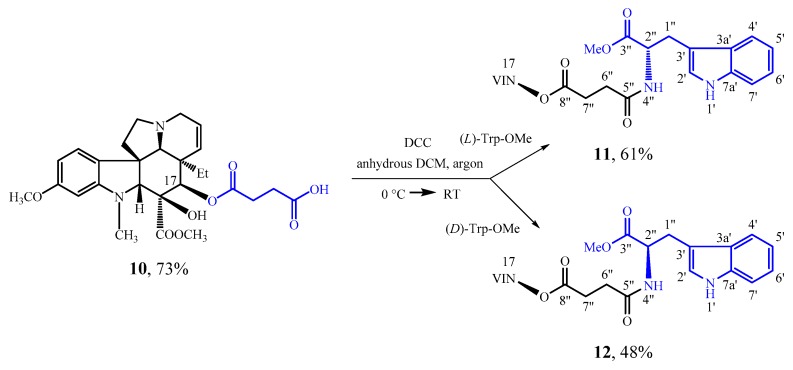
The amidation of compound **10** and (l)- and (d)-tryptophan methyl ester.

The target amino acid-conjugated vindoline derivatives (**11** and **12**) were synthesized from compound **10** [31] derived by the reaction of 17-desacetylvindoline (**9**) [31] with succinic anhydride (Scheme 2). Compound **10** could be coupled with (l)- and (d)-tryptophan methylester (see below). The amidations afforded products **11** and **12** in yields of 61% and 48%, respectively.

#### 2.1.3. Starting from 10-Aminovindoline (5) with Chloroacethyl Chloride 

**Scheme 3:**
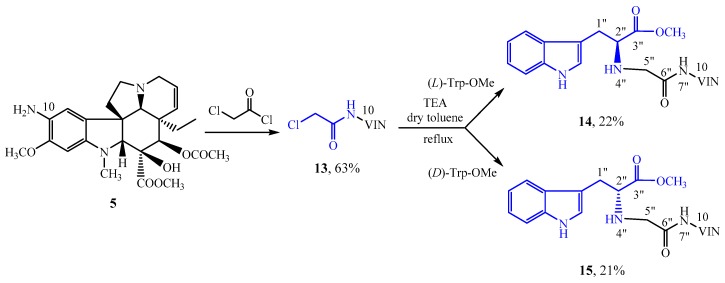
*N*-Alkylation of (l)- and (d)-tryptophan methylester with 10-chloroacetamido-vindoline (**13**).

10-Aminovindoline (**5**) was *N*-acylated with chloroacethyl chloride, giving 10-chloroacethamido-vindoline (**13**, 63%), which was then coupled with (l)- and (d)-tryptophan methylester to afford the expected conjugated derivatives (**14**, **15**) in yields of 22% and 21%, respectively (Scheme 3).

### 2.2. Preparation of Vindoline Derivatives Coupled to a 1,2,3-Triazole Moiety

#### 2.2.1. Click Reaction Using Linker between the Two Pharmacophores

**Scheme 4:**
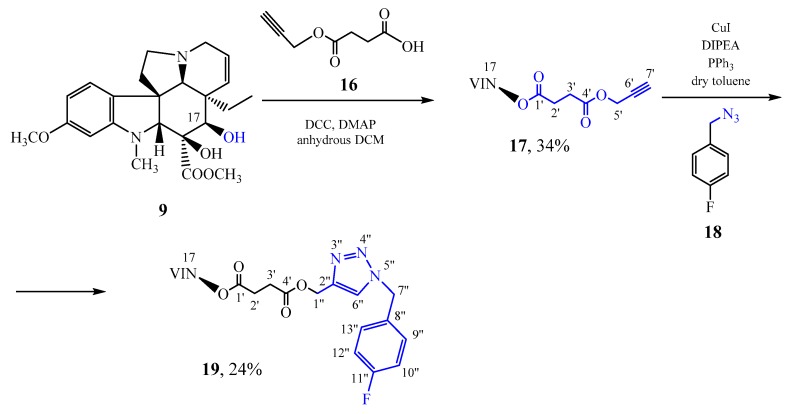
The click reaction of compound **17** and 4-fluorobenzyl-azide (**18**).

The first step was the preparation of the 4-oxo-4-(prop-2-ynyloxy)butanoic acid (**16**) known in the literature [32]. This compound (**16**) could be connected to 17-desacetylvindoline (**9**) in the presence of *N*,*N*’-dicyclohexylcarbodiimide (DCC) and 4-dimethylaminopyridine (DMAP) in dichloromethane (DCM), affording compound **17** in 34% yield. Compound **17** was coupled with 4-fluorobenzyl-azide (**18**) [17] utilizing the widely known copper-catalyzed click chemistry [16]. The reaction was performed in the presence of triphenylphosphine, copper(I) iodide and N,N-diisopropylethylamine (DIPEA) in toluene under reflux to provide the expected conjugated product (**19**) in a yield of 24%, containing a 1,2,3-triazole ring (Scheme 4).

#### 2.2.2. Click Reaction without Linker between the Two Pharmacophores

**Scheme 5:**
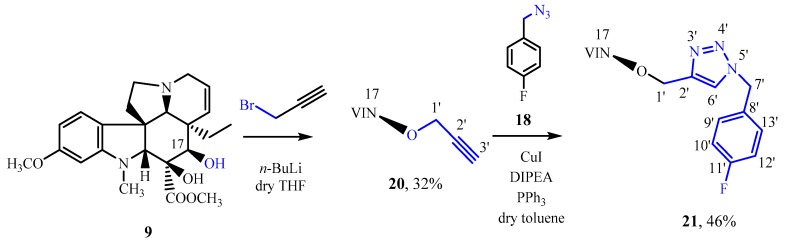
The click reaction of 17-propargylvindoline (**20**) and 4-fluorobenzyl-azide (**18**).

In this reaction, the pharmacophore 1,2,3-triazole derivative was connected to 17-desacetylvindoline (**9**) through only a methylene group. In this case, 17-propargylvindoline (**20**, 32%) had to be synthesized first from propargyl-bromide in the presence of hexamethylphosphoramide and n-buthyllithium in tetrahydrofuran initially at 0 °C, and then by stirring at room temperature. After the click reaction of 17-propargylvindoline (**20)** and 4-fluorobenzyl-azide (**18**) the expected product (**21**) was successfully obtained in a yield of 46% (Scheme 5).

### 2.3. Preparation of Vindoline Derivatives Coupled to Morpholine, Piperazine and N-Methylpiperazine Starting from 17-Desacetylvindoline (**9**)

As a start, 17-desacetylvindoline (**9**) was *O*-acylated with 4-bromobutyric acid, resulting in the formation of the expected 17-(4-bromobutanoyloxy)vindoline (**22**) [22,23]. After this, linker-containing 17-desacetylvindoline derivative (**22**) had been synthesized, the next step was to couple it with the chosen synthetic pharmacophores (morpholine, piperazine and *N*-methylpiperazine).

**Scheme 6:**
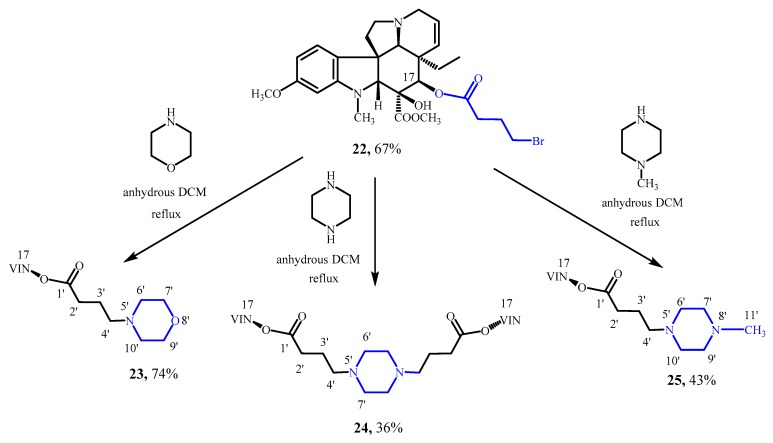
*N*-alkylation of morpholine, piperazine and N-methylpiperazine with 17-(4-bromobutanoyloxy)vindoline (**23**).

First, the coupling reaction with morpholine was studied (Scheme 6). Compound **22** and morpholine was refluxed for 11 h in DCM to afford the expected product (**23**) in a yield of 74%. Using piperazine in DCM provided a dimer product (**24**, 36%) after refluxing for 7 h. (Scheme 6). The preparation of a functionalized derivative only on one side of piperazine (**25**, 43%) was accomplished using *N*-methylpiperazine in DCM after refluxing for 12 h (Scheme 6).

### 2.4. Biology

The compounds were tested for their antiproliferative activity on a set of human gynecological cancer cell lines, such as HeLa and SiHa (cervical), and MCF-7 and MDA-MB-231 (breast); the results are presented in Table 1.

As expected, vindoline (**1**) and 17-desacetylvindoline (**9**) exhibited only inconsiderable anticancer activity on the studied cancer cell lines, while vinblastine (**3**) sulfate showed potent antitumor action with IC_50_ values below 100 nM on most cell lines except for SiHa that was much more resistant to this chemotherapeutic agent (IC_50_ = 14.42 µM). However, a few of the tested *Vinca* derivatives (**11**, **12** and **24**) showed promising results, and this was particularly true concerning their activity on SiHa cells on which vinblastine (**3**) sulfate was the weakest. Compound **12** proved to be more effective than vindoline (**1**) on all cell lines and had an IC_50_ value (12.29 µM) very similar to that of vinblastine (**3**) sulfate (14.42 µM) on SiHa cells. Compound **24** had not only lower IC_50_ values than vindoline (**1**) but proved to be 5-times more effective than vinblastine (**3**) sulfate against SiHa cells. Compound **11** also had a lower IC_50_ value (6.01 µM) than vinblastine (**3**) sulfate on SiHa cells. Of the tested new compounds (not counting references), **24** gave the lowest IC_50_ value (2.85 µM on SiHa cells), and more interestingly, compounds **11**, **12**, and **24** were all more potent than vinblastine (**3**) sulfate on SiHa cells. Accordingly, these compounds demonstrated a dramatic difference compared to vinblastine (**3**) sulfate in their cell line selectivity on HeLa and SiHa cells. While SiHa cells were over 144 times more resistant to vinblastine (**3**) sulfate than HeLa cells, this relative resistance disappeared in the case of compounds **12** and **24**, and even turned to the opposite direction in the case of compound **11** that was at least 5 times more potent against SiHa than HeLa cells. This suggests different biochemical target(s) for these compounds as compared to vinblastine (**3**).

Concerning structure–activity relationships, it seems that the 1,2,3-triazole and morpholine moieties could not improve the efficacy of vindoline (**1**) at all.

To summarize, vindoline (**1**) conjugated with amino acids [(l)- and (d)-tryptophan methyl ester] (compounds **11** and **12**, respectively), piperazine and *N*-methylpiperazine (compounds **24** and **25**, respectively) had significant antiproliferative effect, even though the monomer vindoline (**1**) itself was inactive.

## 3. Experimental Section

### 3.1. General

All the reagents were purchased from Sigma-Aldrich (Budapest, Hungary) and used as received. Melting points were measured on a VEB Analytik Dresden PHMK-77/1328 apparatus (Dresden, Germany) and are uncorrected. IR spectra were recorded on Zeiss IR 75 and 80 instruments (Thornwood, NY, USA). NMR measurements were performed on a Bruker Avance III HDX 400 MHz NMR spectrometer equipped with a ^15^N-^31^P{^1^H-^19^F} 5 mm CryoProbe Prodigy (^31^P: 161.8 MHz), a Bruker Avance III HDX 500 MHz NMR spectrometer equipped with a ^1^H{^13^C/^15^N} 5 mm TCI CryoProbe (^1^H: 499.9 MHz, ^13^C: 125.7 MHz), and a Bruker Avance III HDX 800 MHz NMR spectrometer equipped with a ^1^H-^19^F{^13^C/^15^N} 5 mm TCI CryoProbe (^1^H: 799.7 MHz, ^13^C: 201.1 MHz) (Bruker Corporation, Billerica, MA, USA). ^1^H-^1^H, direct ^1^H-^13^C, and long-range ^1^H-^13^C scalar spin-spin connectivity were established from two-dimensional ^1^H-^1^H correlation spectroscopy (2D COSY), HSQC, and^1^H-^13^C heteronuclear single quantum coherence (HMBC) experiments. ^1^H-^1^H spatial proximities were determined using two-dimensional ^1^H-^1^H nuclear Overhauser effect spectroscopy (NOESY) or ^1^H-^1^H rotating frame nuclear Overhauser effect spectroscopy (ROESY) experiments. ^1^H and ^13^C chemical shifts are given on the delta scale relative to tetramethylsilane. ^15^N chemical shifts were determined on the basis of the ^1^H-^15^N HMBC data and are given on the delta scale relative to nitromethane. All pulse sequences were applied by using the standard spectrometer software package. All experiments were performed at 298 K. NMR spectra were processed using Bruker TopSpin 3.5 pl 7 (Bruker Corporation, Billerica, MA, USA) and ACD/Spectrus Processor version 2017.2.2 (Advanced Chemistry Development, Inc., Toronto, ON, Canada). ESI-HRMS and MS-MS analyses were performed on a Thermo Velos Pro Orbitrap Elite (Thermo Fisher Scientific, Bremen, Germany) system. The ionization method was ESI, operated in positive ion mode. The protonated molecular ion peaks were fragmented by CID (collision-induced dissociation) at a normalized collision energy of 35–45%. For the CID experiment helium was used as the collision gas. The samples were dissolved in methanol. Data acquisition and analysis were accomplished with Xcalibur software version 2.0 (Thermo Fisher Scientific, Bremen, Germany). EI-HRMS analyses were performed on a Thermo Q Exactive GC Orbitrap system. The ionization method was EI and was operated in positive ion mode. The electron energy was 70 eV and the source temperature was set to 250 °C. Data acquisition and analysis were accomplished with Xcalibur software version 4.0 (Thermo Fisher Scientific, Bremen, Germany). TLC was carried out using DC-Alufolien Kieselgel 60 F_254_ (Merck, Budapest, Hungary) plates. Preparative TLC analyses were performed on silica gel 60 PF_254+366_ (Merck, Budapest, Hungary) glass plates. ^1^H and ^13^C NMR data of all the new compounds are available online as a Supplementary Materials.

### 3.2. Synthesis of 10-(3-Carboxypropanamido)vindoline (**6**)

10-Aminovindoline (**5**) [30] (450 mg, 0.95 mmol) and succinic anhydride (144 mg, 1.4 mmol) were dissolved in 20 mL of dry toluene. MS 4 Å molecular sieve was added to the mixture under argon. The reaction mixture was heated to 80 °C and worked up after 32 h. The molecular sieve was filtered and the precipitate remaining on the filter was washed with EtOH. The mixture was evaporated under reduced pressure, then 20 mL of distilled water was added, and the pH was adjusted to 7 with ammonia solution. The aqueous phase was extracted with chloroform (4 × 40 mL). The organic phase was dried over MgSO_4_, then the solvent evaporated under reduced pressure. After preparative TLC (chloroform-methanol = 10:1) 342 mg (63%) of a yellow solid (**6**) was obtained. Mp 147–149 °C. TLC (chloroform-methanol = 10:1); R*_f_* = 0.43. IR (KBr): 3425, 2964, 1742, 1527, 1245, 1039 cm^−1^.

^1^H NMR (399.8 MHz; DMSO-*d*_6_) *δ* (ppm) 0.43 (t; *J* = 7.3 Hz; 3H; H_3_-18); 0.95 (dq; *J* = 13.9; 7.3 Hz; 1H; H_x_-19); 1.47 (dq; *J* = 13.9; 7.3 Hz; 1H; H_y_-19); 1.94 (s; 3H; C(17)-OC(O)CH_3_); 2.17–2.25 (m; 2H; H_2_-6); 2.41–2.48 (br m; 2H; H_2_-3′); 2.50–2.57 (m; 3H; H_x_-5. H_2_-2′); 2.58 (s; 3H; N(1)-CH_3_); 2.59 (s; 1H; H-21); 2.78–2.86 (m; 1H; H_x_-3); 3.24–3.32 (m; 1H; H_y_-5); 3.37–3.44 (m; 1H; H_y_-3); 3.51 (s; 1H; H-2); 3.66 (s; 3H; C(16)-COOCH_3_); 3.80 (s; 3H; C(11)-OCH_3_); 5.06–5.10 (m; 1H; H-15); 5.21 (s; 1H; H-17); 5.82 (ddd; *J* = 10.1; 4.8; 1.1 Hz; 1H; H-14); 6.39 (s; 1H; H-12); 7.57 (s; 1H; H-9); 8.80 (br; 1H; C(16)-OH); 8.92 (s; 1H; C(10)-NH).

^13^C NMR (100.5 MHz; DMSO-*d*_6_) *δ* (ppm) 7.5 (C-18); 20.6 (C(17)-OC(O)CH_3_); 29.1 (C-3′); 30.4 (C-19); 30.7 (C-2′); 38.7 (N(1)-CH_3_); 42.4 (C-20); 43.6 (C-6); 50.3 (C-3); 51.2 (C-5); 51.6 (C(16)-COOCH_3_); 52.3 (C-7); 55.6 (C(11)-OCH_3_); 66.2 (C-21); 75.8 (C-17); 78.6 (C-16); 82.9 (C-2); 93.9 (C-12); 117.5 (C-9); 119.3 (C-10); 123.5 (C-8); 124.4 (C-14); 129.8 (C-15); 149.2 (C-13); 151.1 (C-11); 169.6 (C-1′); 170.0 (C(17)-OC(O)CH_3_); 171.6 (C(16)-COOCH_3_); 173.9 (br; C-4′).

ESI-HRMS: M + H = 572.26097 (delta = 1.3 ppm; C_29_H_38_O_9_N_3_). HR-ESI-MS-MS (CID = 35%; rel. int. %): 554(12); 512(100); 494(3); 480(2); 303(9).

### 3.3. Amidation of 10-(3-Carboxypropanamido)vindoline (**6**) and (l)-Tryptophan Methyl Ester

77 mg (0.35 mmol) of (l)-tryptophan methyl ester was liberated from its hydrochloric acid salt, and dissolved in 10 mL of anhydrous DCM. Under argon at 0 °C, 200 mg (0.35 mmol) of compound **6** was added. Then, 110 mg (0.53 mmol) of DCC was dissolved in 6 mL of anhydrous DCM and added dropwise into the reaction mixture. Subsequently, the mixture was allowed to reach room temperature. After 19 h, the reaction mixture was filtered, and the filtrate was evaporated under reduced pressure. After preparative TLC (dichloromethane-methanol = 10:1) 169 mg (63%) of a pale yellow solid (**7**) was obtained. Mp 140–141 °C. TLC (dichloromethane-methanol = 10:1); R*_f_* = 0.35. IR (KBr): 3370, 2952, 1742, 1525, 1245, 1039, 743 cm^−1^.

^1^H NMR (799.7 MHz; DMSO-*d*_6_) *δ* (ppm) 0.42 (t; *J* = 7.3 Hz; 3H; H_3_-18); 0.94 (dq; *J* = 14.1; 7.3 Hz; 1H; H_x_-19); 1.47 (dq; *J* = 14.1; 7.3 Hz; 1H; H_y_-19); 1.94 (s; 3H; C(17)-OC(O)CH_3_); 2.17–2.23 (m; 2H; H_2_-6); 2.35–2.44 (m; 2H; H_2_-6′’); 2.47–2.53 (m; 3H; H_2_-7′’; H_x_-5); 2.58 (~s; 4H; N(1)-CH_3_; H-21); 2.75–2.79 (m; 1H; H_x_-3); 3.03 (dd; *J* = 14.5; 8.1 Hz; 1H; H_x_-1′’); 3.11 (dd; *J* = 14.5; 6.0 Hz; 1H; H_y_-1′’); 3.25–3.28 (m; 1H; H_y_-5); 3.37–3.41 (m; 1H; H_y_-3); 3.50 (s; 1H; H-2); 3.55 (s; 3H; C(3′’)-OCH_3_); 3.65 (s; 3H; C(16)-COOCH_3_); 3.78 (s; 3H; C(11)-OCH_3_); 4.47–4.51 (m; 1H; H-2′’); 5.06–5.09 (m; 1H; H-15); 5.20 (s; 1H; H-17); 5.81 (ddd; *J* = 10.1; 4.9; 1.1 Hz; 1H; H-14); 6.38 (s; 1H; H-12); 6.97–7.00 (m; 1H; H-5′); 7.05–7.08 (m; 1H; H-6′); 7.16 (d; *J* = 1.6 Hz; 1H; H-2′); 7.33 (d; *J* = 8.1 Hz; 1H; H-7′); 7.48 (d; *J* = 7.8 Hz; 1H; H-4′); 7.53 (s; 1H; H-9); 8.33 (d; *J* = 7.5 Hz; 1H; NH-4′’); 8.78 (s; 1H; C(16)-OH); 8.89 (s; 1H; NH-9′’); 10.85–10.87 (m; 1H; NH-1′).

^13^C NMR (201.1 MHz; DMSO-*d*_6_) *δ* (ppm) 7.5 (C-18); 20.6 (C(17)-OC(O)CH_3_); 27.1 (C-1′’); 30.2 (C-6′’); 30.4 (C-19); 31.0 (C-7′’); 38.7 (N(1)-CH_3_); 42.4 (C-20); 43.6 (C-6); 50.3 (C-3); 51.1 (C-5); 51.6 (C(16)-COOCH_3_; C(3′’)-OCH_3_); 52.3 (C-7); 53.1 (C-2′’); 55.6 (C(11)-OCH_3_); 66.2 (C-21); 75.8 (C-17); 78.6 (C-16); 82.9 (C-2); 93.9 (C-12); 109.3 (C-3′); 111.3 (C-7′); 117.8 (C-9); 117.9 (C-4′); 118.3 (C-5′); 119.1 (C-10); 120.8 (C-6′); 123.4 (C-8); 123.6 (C-2′); 124.3 (C-14); 126.9 (C-3a’); 129.8 (C-15); 135.9 (C-7a’); 149.3 (C-13); 151.3 (C-11); 169.8 (C-8′’); 169.9 (C(17)-OC(O)CH_3_); 171.4 (C-5′’); 171.5 (C(16)-COOCH_3_); 172.3 (C-3′’).

ESI-HRMS: M + H = 772.35419 (delta = −1.3 ppm; C_41_H_50_O_10_N_5_). HR-ESI-MS-MS (CID=35%; rel. int. %): 754(13); 712(100); 694(3); 680(3); 610(2); 554(3); 503(11); 494(3); 472(3); 443(4); 412(4).

### 3.4. Amidation of 10-(3-Carboxypropanamido)vindoline (6) and (d)-Tryptophan Methyl Ester

39 mg (0.18 mmol) of (d)-tryptophan methyl ester was liberated from its hydrochloric acid salt, and dissolved in 5 mL of anhydrous DCM. Under argon at 0 °C, 100 mg (0.18 mmol) of compound **6** was added. Then, 55 mg (0.26 mmol) of N,N’-dicyclohexylcarbodiimide (DCC) was dissolved in 3 mL of anhydrous DCM and added dropwise into the reaction mixture. Subsequently, the mixture was allowed to reach room temperature. After 8 h, the reaction mixture was filtered, and the filtrate was evaporated under reduced pressure. After preparative TLC (dichloromethane-methanol = 10:1) 36 mg (27%) of a pale yellow solid (**8**) was obtained. Mp 152–153 °C. TLC (dichloromethane-methanol = 10:1); R*_f_* = 0.41. IR (KBr): 3308, 2952, 1742, 1525, 1225, 1039, 743 cm^−1^.

^1^H NMR (499.9 MHz; DMSO-*d*_6_) *δ* (ppm) 0.42 (t; *J* = 7.3 Hz; 3H; H_3_-18); 0.95 (dq; *J* = 14.2; 7.3 Hz; 1H; H_x_-19); 1.46 (dq; *J* = 14.2; 7.3 Hz; 1H; H_y_-19); 1.93 (s; 3H; C(17)-OC(O)CH_3_); 2.15–2.24 (m; 2H; H_2_-6); 2.37–2.43 (m; 2H; H_2_-6′’); 2.46–2.54 (m; 3H; H_2_-7′’; H_x_-5); 2.58 (br s; 1H; H-21. s; 3H; N(1)-CH_3_); 2.74–2.79 (m; 1H; H_x_-3); 3.00–3.06 (m; 1H; H_x_-1′’); 3.10–3.15 (m; 1H; H_y_-1′’); 3.24–3.29 (m; 1H; H_y_-5); 3.35–3.42 (m; 1H; H_y_-3); 3.51 (s; 1H; H-2); 3.55 (s; 3H; C(3′’)-OCH_3_); 3.65 (s; 3H; C(16)-COOCH_3_); 3.78 (s; 3H; C(11)-OCH_3_); 4.47–4.53 (m; 1H; H-2′’); 5.05–5.09 (m; 1H; H-15); 5.20 (s; 1H; H-17); 5.81 (ddd; *J* = 10.1; 4.9; 1.5 Hz; 1H; H-14); 6.38 (s; 1H; H-12); 6.96–7.00 (m; 1H; H-5′); 7.04–7.08 (m; 1H; H-6′); 7.16 (d; *J* = 2.2 Hz; 1H; H-2′); 7.31–7.35 (m; 1H; H-7′); 7.46–7.50 (m; 1H; H-4′); 7.54 (s; 1H; H-9); 8.32 (d; *J* = 7.5 Hz; 1H; NH-4′’); 8.78 (br s; 1H; C(16)-OH); 8.88 (s; 1H; NH-9′’); 10.86 (br d; *J* = 2.2 Hz; 1H; NH-1′).

^13^C NMR (125.7 MHz; DMSO-*d*_6_) *δ* (ppm) 7.5 (C-18); 20.6 (C(17)-OC(O)CH_3_); 27.1 (C-1′’); 30.2 (C-6′’); 30.4 (C-19); 31.1 (C-7′’); 38.7 (N(1)-CH_3_); 42.4 (C-20); 43.6 (C-6); 50.3 (C-3); 51.1 (C-5); 51.6 (C(3′’)-OCH_3_. C(16)-COOCH_3_); 52.3 (C-7); 53.1 (C-2′’); 55.6 (C(11)-OCH_3_); 66.2 (C-21); 75.8 (C-17); 78.6 (C-16); 82.9 (C-2); 93.9 (C-12); 109.3 (C-3′); 111.3 (C-7′); 117.8 (C-9); 117.9 (C-4′); 118.3 (C-5′); 119.2 (C-10); 120.8 (C-6′); 123.5 (C-8); 123.6 (C-2′); 124.4 (C-14); 127.0 (C-3a’); 129.8 (C-15); 136.0 (C-7a’); 149.3 (C-13); 151.3 (C-11); 169.8 (C-8′’); 170.0 (C(17)-OC(O)CH_3_); 171.4 (C-5′’); 171.5 (C(16)-COOCH_3_); 172.3 (C-3′’).

ESI-HRMS: M + H = 772.35387 (delta = −1.75 ppm; C_41_H_50_O_10_N_5_). HR-ESI-MS-MS (CID=35%; rel. int. %): 754(15); 712(100); 694(3); 680(3); 610(2); 554(3); 503(13); 494(4); 472(4); 443(6); 412(5).

### 3.5. Amidation of 17-(3-Carboxypropanoyloxy)vindoline (**10**) and (l)-Tryptophan Methyl Ester

85 mg (0.39 mmol) of (l)-tryptophan methyl ester was liberated from its hydrochloric acid salt, and dissolved in 10 mL of anhydrous DCM. Under argon the mixture was cooled to 0 °C, and 200 mg (0,39 mmol) of compound **10** [31] was added. Then, 82 mg (0.39 mmol) of DCC dissolved in 5 mL anhydrous DCM was added dropwise into the reaction mixture. Then, the mixture was allowed to reach room temperature. After stirring for 22 h, the precipitate was filtered, and the filtrate was evaporated under reduced pressure. After preparative TLC (dichloromethane-methanol = 10:1) 170 mg (61%) of a pale yellow solid (**11**) was obtained. Mp 131–133 °C. TLC (dichloromethane-methanol = 20:1); R*_f_* = 0.34. IR (KBr): 3369, 2952, 1741, 1502, 1225, 1168, 1027, 744 cm^−1^.

^1^H NMR (499.9 MHz; DMSO-*d*_6_) *δ* (ppm) 0.41 (t; *J* = 7.3 Hz; 3H; H_3_-18); 0.92 (dq; *J* = 14.1; 7.3 Hz; 1H; H_x_-19); 1.46 (dq; *J* = 14.1; 7.3 Hz; 1H; H_y_-19); 2.14–2.24 (m. 2H; H_2_-6); 2.27–2.45 (m; 4H; H_2_-6′’. H_2_-7′’); 2.52–2.59 (m; 1H; H_x_-5); 2.56 (s; 3H; N(1)-CH_3_); 2.65 (s; 1H; H-21); 2.74–2.80 (m; 1H; H_x_-3); 2.99–3.06 (m; 1H; H_x_-1′’); 3.10–3.15 (m; 1H; H_y_-1′’); 3.24–3.30 (m; 1H; H_y_-5); 3.35–3.41 (m; 1H; H_y_-3); 3.54 (~s; 4H; H-2. C(3′’)-OCH_3_); 3.63 (s; 3H; C(16)-COOCH_3_); 3.70 (s; 3H; C(11)-OCH_3_); 4.45–4.50 (m; 1H; H-2′’); 5.11–5.15 (m; 1H; H-15); 5.18 (s; 1H; H-17); 5.75 (ddd; *J* = 10.2; 4.8; 1.3 Hz; 1H; H-14); 6.19 (d; *J* = 2.2 Hz; 1H; H-12); 6.28 (dd; *J* = 8.2; 2.2 Hz; 1H; H-10); 6.96–7.00 (m; 1H; H-5′); 7.04 (d; *J* = 8.2 Hz; 1H; H-9); 7.04–7.08 (m; 1H; H-6′); 7.14 (d; *J* = 2.3 Hz; 1H; H-2′); 7.32–7.35 (m; 1H; H-7′); 7.46–7.49 (m; 1H; H-4′); 8.36 (d; *J* = 7.5 Hz; 1H; NH-4′’); 8.80 (br s; 1H; C(16)-OH); 10.86 (br d; *J* = 2.3 Hz; 1H; NH-1′).

^13^C NMR (125.7 MHz; DMSO-*d*_6_) *δ* (ppm) 7.5 (C-18); 27.0 (C-1′’); 28.9. 29.6 (C-6′’. C-7′’); 30.3 (C-19); 38.0 (N(1)-CH_3_); 42.4 (C-20); 43.7 (C-6); 50.3 (C-3); 51.0 (C-5); 51.6 (C(3′’)-OCH_3_); 51.7 (C(16)-COOCH_3_); 52.0 (C-7); 53.2 (C-2′’); 55.0 (C(11)-OCH_3_); 66.0 (C-21); 75.8 (C-17); 78.6 (C-16); 82.8 (C-2); 95.4 (C-12); 104.4 (C-10); 109.3 (C-3′); 111.3 (C-7′); 117.9 (C-4′); 118.3 (C-5′); 120.9 (C-6′); 123.0 (C-9); 123.6 (C-2′); 124.1 (C-14); 125.3 (C-8); 127.0 (C-3a’); 129.9 (C-15); 136.0 (C-7a’); 153.4 (C-13); 160.4 (C-11); 170.7 (C-5′’); 171.5 (C(16)-COOCH_3_); 171.8 (C-8′’); 172.3 (C-3′’).

^15^N NMR (50.7 MHz; DMSO-*d*_6_) *δ* (ppm) −323.2 (N-4); -315.0 (N-1); −262.2 (N-4′’); −250.8 (N-1′).

ESI-HRMS: M + H = 715.33393 (delta = 0.24 ppm; C_39_H_47_O_9_N_4_). HR-ESI-MS-MS (CID = 35%; rel. int. %): 697(100); 415(6); 397(9).

### 3.6. Amidation of 17-(3-Carboxypropanoyloxy)vindoline (**10**) and (d)-Tryptophan Methyl Ester

85 mg (0.39 mmol) of (d)-tryptophan methyl ester was liberated from its hydrochloric acid salt, and dissolved in 10 mL of anhydrous DCM. Under argon the mixture was cooled to 0 °C, and 200 mg (0,39 mmol) of compound **10** [31] was added. Then, 82 mg (0.39 mmol) of DCC dissolved in 5 mL of anhydrous DCM was added dropwise into the reaction mixture. Then, the mixture was allowed to reach room temperature. After stirring for 22 h, the precipitate was filtered, and the filtrate was evaporated under reduced pressure. After preparative TLC (dichloromethane-methanol = 15:1) 133 mg (48%) of a pale yellow solid (**12**) was obtained. Mp 141–143 °C. TLC (dichloromethane-methanol = 15:1); R*_f_* = 0.54. IR (KBr): 3380, 2952, 1741, 1502, 1225, 1168, 1028, 744 cm^−1^.

^1^H NMR (499.9 MHz; DMSO-*d*_6_) *δ* (ppm) 0.40 (t; *J* = 7.4 Hz; 3H; H_3_-18); 0.92 (dq; *J* = 14.3; 7.4 Hz; 1H; H_x_-19); 1.46 (dq; *J* = 14.3; 7.4 Hz; 1H; H_y_-19); 2.14–2.24 (m. 2H; H_2_-6); 2.27–2.45 (m; 4H; H_2_-6′’. H_2_-7′’); 2.52–2.59 (m; 1H; H_x_-5); 2.56 (s; 3H; N(1)-CH_3_); 2.65 (s; 1H; H-21); 2.75–2.82 (m; 1H; H_x_-3); 2.98–3.05 (m; 1H; H_x_-1′’); 3.08–3.14 (m; 1H; H_y_-1′’); 3.24–3.30 (m; 1H; H_y_-5); 3.38–3.44 (m; 1H; H_y_-3); 3.54 (s; 1H; H-2); 3.55 (s; 3H; C(3′’)-OCH_3_); 3.63 (s; 3H; C(16)-COOCH_3_); 3.70 (s; 3H; C(11)-OCH_3_); 4.46–4.52 (m; 1H; H-2′’); 5.12–5.16 (m; 1H; H-15); 5.19 (s; 1H; H-17); 5.81 (ddd; *J* = 10.3; 4.8; 1.3 Hz; 1H; H-14); 6.18 (d; *J* = 2.3 Hz; 1H; H-12); 6.28 (dd; *J* = 8.2; 2.3 Hz; 1H; H-10); 6.94–6.98 (m; 1H; H-5′); 7.03–7.07 (m; 1H; H-6′); 7.04 (d; *J* = 8.2 Hz; 1H; H-9); 7.13 (d; *J* = 2.3 Hz; 1H; H-2′); 7.31–7.34 (m; 1H; H-7′); 7.46–7.48 (m; 1H; H-4′); 8.35 (d; *J* = 7.5 Hz; 1H; NH-4′’); 8.79 (br s; 1H; C(16)-OH); 10.85 (br d; *J* = 2.3 Hz; 1H; NH-1′).

^13^C NMR (125.7 MHz; DMSO-*d*_6_) *δ* (ppm) 7.5 (C-18); 27.0 (C-1′’); 28.9. 29.6 (C-6′’. C-7′’); 30.3 (C-19); 38.0 (N(1)-CH_3_); 42.4 (C-20); 43.7 (C-6); 50.3 (C-3); 51.0 (C-5); 51.6 (C(3′’)-OCH_3_); 51.7 (C(16)-COOCH_3_); 52.0 (C-7); 53.1 (C-2′’); 55.0 (C(11)-OCH_3_); 66.1 (C-21); 75.8 (C-17); 78.6 (C-16); 82.8 (C-2); 95.4 (C-12); 104.4 (C-10); 109.3 (C-3′); 111.3 (C-7′); 117.9 (C-4′); 118.3 (C-5′); 120.8 (C-6′); 123.0 (C-9); 123.5 (C-2′); 124.1 (C-14); 125.3 (C-8); 127.0 (C-3a’); 130.0 (C-15); 136.0 (C-7a’); 153.4 (C-13); 160.4 (C-11); 170.7 (C-5′’); 171.6 (C(16)-COOCH_3_); 171.8 (C-8′’); 172.3 (C-3′’).

ESI-HRMS: M + H = 715.33370 (delta = −0.08 ppm; C_39_H_47_O_9_N_4_). HR-ESI-MS-MS (CID = 45%; rel. int. %): 697(100); 415(5); 397(9).

### 3.7. N-Acylation of 10-Aminovindoline (**5**) with Chloroacetyl Chloride

978 mg (2.1 mmol) of 10-aminovindoline (**5**) [30] was dissolved in 20 mL of anhydrous DCM under argon, then 321 mg (2.3 mmol) of anhydrous K_2_CO_3_ was added. The reaction mixture was cooled to 0 °C, and 1.71 mL (21.5 mmol) of chloroacetyl chloride was added dropwise. The mixture was allowed to reach room temperature. After stirring for 6 h, the reaction mixture was filtered, and the filtrate was washed with 50 mL of 10% NaHCO_3_ solution. The aqueous phase was extracted with 2 × 50 mL DCM. The organic phase was dried over MgSO_4_, then the solvent evaporated under reduced pressure. After preparative TLC (ethyl acetate: methanol = 15:1) 712 mg (63%) of a white crystalline pure product (**13**) was obtained. Mp 168 °C. TLC (ethyl acetate: methanol = 20:1); R*_f_* = 0.30. IR (KBr): 3392, 2964, 1743,1533, 1245, 1039 cm^−1^.

^1^H NMR (499.9 MHz; CDCl_3_) *δ* (ppm) 0.53 (3H; t; *J* = 7.3 Hz; H_3_-18); 1.08 (1H; dq; *J* = 14.3; 7.3 Hz; H_x_-19); 1.62 (1H; dq; *J* = 14.3; 7.3 Hz; H_y_-19); 2.08 (3H; s; C(17)-OC(O)CH_3_); 2.25–2.40 (2H; m; H_2_-6); 2.54–2.63 (1H; m; H_x_-5); 2.69 (3H; s; N(1)-CH_3_); 2.77 (1H; s; H-21); 2.81–2.89 (1H; m; H_x_-3); 3.36–3.45 (1H; m; H_y_-5); 3.45–3.54 (1H; m; H_y_-3); 3.73 (1H; s; H-2); 3.79 (3H; s; C(16)-COOCH_3_); 3.91 (3H; s; C(11)-OCH_3_); 4.16 (2H; ~s; C(10)-NH-C(O)CH_2_Cl); 5.24 (1H; br d; *J* = 10.4 Hz; H-15); 5.43 (1H; s; H-17); 5.86 (1H; ddd; *J* = 10.4; 5.0; 1.4 Hz; H-14); 6.13 (1H; s; H-12); 8.04 (1H; s; H-9); 8.72 (1H; br; C(10)-NH); 9.60 (1H; br; C(16)-OH).

^13^C NMR (201.1 MHz; CDCl_3_) *δ* (ppm) 7.6 (C-18); 21.1 (C(17)-OC(O)CH_3_); 31.0 (C-19); 38.9 (N(1)-CH_3_); 42.9 (C-20); 43.1 (C(10)-NH-C(O)CH_2_Cl); 43.9 (br; C-6); 50.9 (C-3); 51.5 (br; C-5); 52.3 (C(16)-COOCH_3_); 53.3 (br; C-7); 56.1 (C(11)-OCH_3_); 66.5 (br; C-21); 76.4 (C-17); 79.7 (C-16); 83.4 (br; C-2); 93.2 (C-12); 114.5 (C-9); 119.0 (C-10); 123.6 (br; C-8); 124.2 (br; C-14); 130.4 (C-15); 149.5 (C-13); 149.9 (C-11); 163.0 (C(10)-NH-C(O)CH_2_Cl); 170.8 (C(17)-OC(O)CH_3_); 171.8 (C(16)-COOCH_3_).

ESI-HRMS: M + H = 548.21557 (delta = −0.4 ppm; C_27_H_35_O_7_N_3_Cl). HR-ESI-MS-MS (CID = 35%. rel. int. %): 530(11); 488(100); 470(2);456(3); 428(2); 386(1); 279(7).

### 3.8. N-Alkylation of (l)-Tryptophan Methyl Ester with 10-Chloroacetamido-Vindoline (**13**)

10-Chloroacetamido-vindoline (**13**) (200 mg, 0.37 mmol) and (l)-tryptophan methyl ester (88 mg, 0.40 mmol; previously liberated from its hydrochloric acid salt) were dissolved in 5 mL of dry toluene in a sealed glass. Then, 0.05 mL (0.37 mmol) of TEA was added. The sealed glass was placed in an oil bath at 150 °C. After stirring for 15 h, the precipitate was filtered, and the filtrate was evaporated under reduced pressure. After preparative TLC (dichloromethane-methanol = 10:1) 59 mg (22%) of a pale green solid (**14**) was obtained. Mp 150–152 °C. TLC (dichloromethane-methanol = 10:1); R*_f_* = 0.54. IR (KBr): 3319, 2950, 1740, 1528, 1459, 1243, 1039, 743 cm^−1^.

^1^H NMR (499.9 MHz; DMSO-*d*_6_) *δ* (ppm) 0.42 (t; *J* = 7.3 Hz; 3H; H_3_-18); 0.96 (dq; *J* = 13.8; 7.3 Hz; 1H; H_x_-19); 1.49 (dq; *J* = 13.8; 7.3 Hz; 1H; H_y_-19); 1.95 (s; 3H; C(17)-OC(O)CH_3_); 2.17–2.28 (m; 2H; H_2_-6); 2.52–2.60 (m; 1H; H_x_-5); 2.60 (s; 3H; N(1)-CH_3_); 2.65 (br s; 1H; H-21); 2.81–2.90 (m; 1H; H_x_-3); 3.08–3.19 (m; 3H; H_2_-1′’. H_x_-5′’); 3.31–3.41 (m; 2H; H_y_-5. H_y_-5′’); 3.41–3.48 (m; 1H; H_y_-3); 3.53 (s; 1H; H-2); 3.55 (s; 3H; C(3′’)-OCH_3_); 3.58–3.62 (m; 1H; H-2′’); 3.66 (s; 3H; C(16)-COOCH_3_); 3.82 (s; 3H; C(11)-OCH_3_); 5.08–5.12 (m; 1H; H-15); 5.21 (s; 1H; H-17); 5.83 (ddd; *J* = 10.1; 4.8; 1.5 Hz; 1H; H-14); 6.45 (s; 1H; H-12); 6.93–6.97 (m; 1H; H-5′); 7.04–7.07 (m; 1H; H-6′); 7.14 (d; *J* = 2.2 Hz; 1H; H-2′); 7.32–7.35 (m; 1H; H-7′); 7.47–7.50 (m; 1H; H-4′); 7.91 (s; 1H; H-9); 8.79 (br s; 1H; C(16)-OH); 9.49 (s; 1H; NH-7′’); 10.89 (br d; *J* = 2.2 Hz; 1H; NH-1′).

^13^C NMR (125.7 MHz; DMSO-*d*_6_) *δ* (ppm): 7.4 (C-18). 20.6 (C(17)-OC(O)CH_3_). 28.5 (C-1′’). 30.4 (C-19). 38.8 (N(1)-CH_3_). 42.4 (C-20). 43.5 (C-6). 50.3 (C-3). 50.8 (C-5′’). 51.1 (C-5). 51.4 (C(3′’)-OCH_3_). 51.7 (C(16)-COOCH_3_). 52.4 (C-7). 55.9 (C(11)-OCH_3_). 61.7 (C-2′’). 66.2 (C-21). 75.7 (C-17). 78.6 (C-16). 82.6 (C-2). 94.0 (C-12). 109.2 (C-3′). 111.3 (C-7′). 114.5 (C-9). 117.9 (C-4′). 118.2 (C-5′). 119.3 (C-10). 120.8 (C-6′). 123.4 (C-8). 123.5 (C-2′). 124.3 (C-14). 127.1 (C-3a’). 129.8 (C-15). 136.0 (C-7a’). 148.7 (C-13). 149.8 (C-11). 168.3 (br; C-6′’). 170.0 (C(17)-OC(O)CH_3_). 171.5 (C(16)-COOCH_3_). 173.8 (br; C-3′’).

ESI-HRMS: M + H = 730.34419 (delta = −0.64 ppm; C_39_H_48_O_9_N_5_). HR-ESI-MS-MS (CID = 35%; rel. int. %): 712(12); 670(100); 652(3); 568(4); 461(5); 433(3); 346(3).

### 3.9. N-Alkylation of (d)-Tryptophan Methyl Ester with 10-Chloroacetamido-Vindoline (**13**)

10-Chloroacetamido-vindoline (**13**) (200 mg, 0.37 mmol) and (d)-tryptophan methyl ester (88 mg, 0.40 mmol; previously liberated from its hydrochloric acid salt) were dissolved in 5 mL of dry toluene in a sealed glass. Then, 0.05 mL (0.37 mmol) of TEA was added. The sealed glass was placed in an oil bath at 150 °C. After stirring for 12 h, the precipitate was filtered, and the filtrate was evaporated under reduced pressure. After preparative TLC (dichloromethane-methanol = 10: 1) 56 mg (21%) of a pale green solid (**15**) was obtained. Mp 157–159 °C. TLC (dichloromethane-methanol = 10:1); R*_f_* = 0.58. IR (KBr): 3325, 2950, 1740, 1528, 1459, 1244, 1039, 743 cm^−1^.

^1^H NMR (499.9 MHz; DMSO-*d*_6_) *δ* (ppm) 0.42 (t; *J* = 7.3 Hz; 3H; H_3_-18); 0.94 (dq; *J* =14.1; 7.3 Hz; 1H; H_x_-19); 1.47 (dq; *J* =14.1; 7.3 Hz; 1H; H_y_-19); 1.94 (s; 3H; C(17)-OC(O)CH_3_); 2.16–2.27 (m; 2H; H_2_-6); 2.50–2.56 (m; 1H; H_x_-5); 2.60 (~s; 4H; N(1)-CH_3_. H-21); 2.79–2.85 (m; 1H; H_x_-3); 2.92–3.11 (br m; 1H; NH-4′’); 3.07–3.12 (m; 3H; H_x_-5′’. H_2_-1′’); 3.28–3.38 (m; 2H; H_y_-5. H_y_-5′’); 3.39–3.45 (m; 1H; H_y_-3); 3.52 (s; 1H; H-2); 3.55 (s; 3H; C(3′’)-OCH_3_); 3.55–3.60 (m; 1H; H-2′’); 3.66 (s; 3H; C(16)-COOCH_3_); 3.82 (s; 3H; C(11)-OCH_3_); 5.07–5.11 (m; 1H; H-15); 5.20 (s; 1H; H-17); 5.83 (ddd; *J* = 10.4; 4.2; 1.1 Hz; 1H; H-14); 6.45 (s; 1H; H-12); 6.94–6.98 (m; 1H; H-5′); 7.03–7.07 (m; 1H; H-6′); 7.14 (d; *J* = 2.0 Hz; 1H; H-2′); 7.31–7.35 (m; 1H; H-7′); 7.47–7.51 (m; 1H; H-4′); 7.91 (s; 1H; H-9); 8.77 (br s; 1H; C(16)-OH); 9.48 (s; 1H; NH-7′’); 10.88 (br d; *J* = 2.0 Hz; 1H; NH-1′).

^13^C NMR (125.7 MHz; DMSO-*d*_6_) *δ* (ppm) 7.5 (C-18); 20.7 (C(17)-OC(O)CH_3_); 28.6 (C-1′’); 30.4 (C-19); 38.8 (N(1)-CH_3_); 42.4 (C-20); 43.6 (C-6); 50.4 (C-3); 51.0 (C-5′’); 51.1 (C-5); 51.4 (C(3′’)-OCH_3_); 51.6 (C(16)-COOCH_3_); 52.5 (C-7); 55.9 (C(11)-OCH_3_); 61.8 (C-2′’); 66.1 (C-21); 75.8 (C-17); 78.7 (C-16); 82.8 (C-2); 94.0 (C-12); 109.3 (C-3′); 111.3 (C-7′); 114.3 (C-9); 117.9 (C-4′); 118.3 (C-5′); 119.4 (C-10); 120.8 (C-6′); 123.4 (C-8); 123.5 (C-2′); 124.4 (C-14); 127.1 (C-3a’); 129.8 (C-15); 136.0 (C-7a’); 148.6 (C-13); 149.7 (C-11); 168.4 (C-6′’); 170.0 (C(17)-OC(O)CH_3_); 171.5 (C(16)-COOCH_3_); 174.0 (C-3′’).

ESI-HRMS: M+H=730.34353 (delta=-1.54 ppm; C_39_H_48_O_9_N_5_). HR-ESI-MS-MS (CID=35%; rel. int. %): 712(13); 670(100); 652(3); 568(4); 461(7); 433(4); 346(4).

### 3.10. Synthesis of 17-[4-Oxo-4-(prop-2-ynyloxy)butanoyloxy]vindoline (**17**)

17-Desacetylvindoline (**9**) [31] (600 mg, 1.5 mmol) and 4-oxo-4- (prop-2-ynyloxy) butanoic acid (**16**) (240 mg, 1.5 mmol) were dissolved in 15 mL of anhydrous DCM followed by a dropwise addition of 330 mg (0.53 mmol) of DCC and 15 mg (0.039 mmol) of 4-dimethylaminopyridine (DMAP) dissolved in another 15 mL of anhydrous DCM. The reaction mixture was refluxed for 4h, then further 250 mg of DCC and 30 mg of DMAP dissolved in 20 mL of anhydrous DCM were added in two portions. After refluxing for 46 h, the reaction mixture was filtered, and the filtrate was evaporated under reduced pressure. After preparative TLC (dichloromethane: methanol = 10: 1) 269 mg (34%) of a pale brown crystalline product (**17**) was obtained. Mp 123–130 °C. TLC (dichloromethane: methanol = 10: 1); R*_f_* = 0.75. IR (KBr): 1156; 1223; 1262; 1619; 1732; 2944; 3258 cm^−1^.

^1^H NMR (499.9 MHz; DMSO-*d*_6_) *δ* (ppm) 0.42 (t; *J* = 7.4 Hz; 3H; H_3_-18); 0.94 (dq; *J* = 14.0. 7.4 Hz; 1H; H_x_-19); 1.46 (dq; *J* = 14.0. 7.4 Hz; 1H; H_y_-19); 2.17–2.25 (m; 2H; H_2_-6); 2.41–2.60 (m; 8H; H_2_-2′. H_2_-3′. N(1)-CH_3_. H_x_-5); 2.66 (s; 1H; H-21); 2.79 (ddd; *J* = 16.2. 2.7. 1.5 Hz; 1H; H_x_-3); 3.25–3.31 (m; 1H; H_y_-5); 3.42 (ddd; *J* = 16.2. 4.9. 1.2 Hz; 1H; H_y_-3); 3.54 (t; *J* = 2.5 Hz; 1H; H-7′); 3.55 (s; 1H; H-2); 3.65 (s; 3H; C(16)-COOCH_3_); 3.70 (s; 3H; C(11)-OCH_3_); 4.64–4.72 (m; 2H; H_2_-5′); 5.13 (ddd; *J* = 10.2. 2.7. 1.2 Hz; 1H; H-15); 5.20 (s; 1H; H-17); 5.83 (ddd; *J* = 10.2. 4.9. 1.5 Hz; 1H; H-14); 6.19 (d; *J* = 2.3 Hz; 1H; H-12); 6.28 (dd; *J* = 8.2. 2.3 Hz; 1H; H-10); 7.05 (d; *J* = 8.2 Hz; 1H; H-9); 8.79 (s; 1H; C(16)-OH).

^13^C NMR (125.7 MHz; DMSO-*d*_6_) *δ* (ppm) 7.5 (C-18); 28.3 (C-3′); 28.5 (C-2′); 30.3 (C-19); 38.0 (N(1)-CH_3_); 42.4 (C-20); 43.6 (C-6); 50.3 (C-3); 51.0 (C-5); 51.70 (C(16)-COOCH_3_); 51.73 (C-5′); 52.0 (C-7); 55.0 (C(11)-OCH_3_); 66.1 (C-21); 76.1 (C-17); 77.6 (C-7′); 78.2 (C-6′); 78.6 (C-16); 82.8 (C-2); 95.4 (C-12); 104.5 (C-10); 123.0 (C-9); 124.3 (C-14); 125.3 (C-8); 129.8 (C-15); 153.4 (C-13); 160.4 (C-11); 171.1 (C-4′); 171.3 (C-1′); 171.5 (C(16)-COOCH_3_).

^15^N NMR (50.7 MHz; DMSO-*d*_6_) *δ* (ppm) −322.4 (N-4); −314.2 (N-1).

ESI-HRMS: M + H = 553.25395 (delta = −0.9ppm; C_30_H_37_O_8_N_2_). HR-ESI-MS-MS (CID = 35%; rel. int. %): 535(11); 493(13); 397(100); 365(4); 188(25).

### 3.11. The Click Reaction of 17-[4-Oxo-4-(prop-2-ynyloxy)butanoyloxy]vindoline (**17**) and 4-Fluorobenzyl-Azide (**18**)

299 mg (0.54 mmol) of 17-[4-oxo-4-(prop-2-ynyloxy)butanoyloxy]vindoline (**17**), 86 mg (0.57 mmol) of 4-fluorobenzyl-azide (**18**), 29 mg (0.11 mmol) of triphenylphosphine and 11 mg (0.054 mmol) of copper (I) iodide were dissolved in 12 mL of dry toluene, then 0.29 mL (1.6 mmol) of *N*,*N*-diisopropylethylamine (DIPEA) was added, and the reaction mixture was refluxed for 4 h. Then, 75 mL of toluene was added and the mixture was washed with 90 mL of distilled water, finally the aqueous phase was washed with another 30 mL of toluene. The organic phase was dried over MgSO_4_, then the solvent evaporated under reduced pressure. After preparative TLC (dichloromethane-methanol = 30:1 then dichloromethane-methanol = 15:1) 90 mg (24%) of a white crystalline product (**19**) was obtained. Mp 80–87 °C. TLC (dichloromethane-methanol = 15:1); R*_f_* = 0,72. IR (KBr): 1158; 1225; 1503; 1738; 2838; 2962 cm^−1^.

^1^H NMR (499.9 MHz; DMSO-*d*_6_) *δ* (ppm) 0.41 (t; *J* = 7.4 Hz; 3H; H_3_-18); 0.93 (dq; *J* = 13.9. 7.4 Hz; 1H; H_x_-19); 1.44 (dq; *J* = 13.9. 7.4 Hz; 1H; H_y_-19); 2.17–2.25 (m; 2H; H_2_-6); 2.39–2.61 (m; 8H; H_2_-3′. H_2_-2′. N(1)-CH_3_. H_x_-5); 2.66 (s; 1H; H-21); 2.79 (br d; *J* = 16.1 Hz; 1H; H_x_-3); 3.25–3.31 (m; 1H; H_y_-5); 3.40 (br dd; *J* = 16.1. 4.9 Hz; 1H; H_y_-3); 3.55 (s; 1H; H-2); 3.63 (s; 3H; C(16)-COOCH_3_); 3.70 (s; 3H; C(11)-OCH_3_); 5.08–5.12 (m; 1H; H-15), 5.11 (s; 2H; H_2_-1′’); 5.20 (s; 1H; H-17); 5.58 (s; 2H; H_2_-7′’); 5.79 (ddd; *J* = 10.2. 4.9. 1.4 Hz; 1H; H-14); 6.19 (d; *J* = 2.2 Hz; 1H; H-12); 6.28 (dd; *J* = 8.2. 2.2 Hz; 1H; H-10); 7.05 (d; *J* = 8.2 Hz; 1H; H-9); 7.17–7.23 (m; 2H; H-10′’. H-12′’); 7.36–7.42 (m; 2H; H-9′’. H-13′’); 8.19 (s; 1H; H-6′’); 8.80 (s; 1H; C(16)-OH).

^13^C NMR (125.7 MHz; DMSO-*d*_6_) *δ* (ppm) 7.5 (C-18); 28.4 (C-2′); 28.5 (C-3′); 30.4 (C-19); 38.0 (N(1)-CH_3_); 42.4 (C-20); 43.6 (C-6); 50.3 (C-3); 51.1 (C-5); 51.7 (C(16)-COOCH_3_); 51.9 (C-7′’); 52.0 (C-7); 55.0 (C(11)-OCH_3_); 57.3 (C-1′’); 66.1 (C-21); 76.1 (C-17); 78.6 (C-16); 82.7 (C-2); 95.4 (C-12); 104.5 (C-10); 115.5 (d; ^2^*J*_FC_ = 21.6 Hz; C-10′’. C-12′’); 123.0 (C-9); 124.2 (C-14); 124.6 (C-6′’); 125.3 (C-8); 129.8 (C-15); 130.3 (d; ^3^*J*_FC_ = 8.4 Hz; C-9′’. C-13′’); 132.1 (d; ^4^*J*_FC_ = 3.1 Hz; C-8′’); 142.0 (C-2′’); 153.4 (C-13); 160.5 (C-11); 161.8 (d; ^1^*J*_FC_ = 244.4 Hz; C-11′’); 171.4 (C-1′); 171.6 (C-4′. C(16)-COOCH_3_).

^15^N NMR (50.7 MHz; DMSO-*d*_6_) *δ* (ppm) −322.8 (N-4); −314.4 (N-1); −130.8 (N-5′’); −27.7 (N-3′’); −18.7 (N-4′’).

ESI-HRMS: M + H = 704.30669 (delta = −3.3ppm; C_37_H_43_O_8_N_5_F). HR-ESI-MS-MS (CID = 35%; rel. int. %): 686(100); 644(5); 397(22).

### 3.12. Synthesis of 17-Propargylvindoline (**20**)

17-Desacetylvindoline (**9**) [31] (600 mg, 1.4 mmol) and hexamethylphosphoramide (0.25 mL, 1.5 mmol) were dissolved in 18 mL of dry tetrahydrofuran under argon. The reaction mixture was cooled to 0 °C, and a solution of 0.60 mL (1.6 mmol) 2.5 M n-butyllithium in hexane was added dropwise via a syringe. After stirring for 5 min at 0 °C, propargyl bromide (0.156 mL, 1.4 mmol) was added, while maintaining the temperature at 0 ° C. After stirring for 16 h at room temperature, the mixture was diluted with 30 mL of distilled water and 60 mL of 10% Na_2_CO_3_ solution, then it was extracted with dichloromethane (3 × 60 mL). The organic phase was dried over MgSO_4_, then the solvent evaporated under reduced pressure. After preparative TLC (dichloromethane-methanol = 20:1) 185 mg (32%) of a brown crystalline product (**20**) was obtained. Mp 80–88 °C. TLC (dichloromethane-methanol = 20:1); R*_f_* = 0,33. IR (KBr): 1080; 1245; 1502; 1735; 2931; 3276 cm^−1^.

^1^H NMR (499.9 MHz; DMSO-*d*_6_) *δ* (ppm) 0.49 (t; *J* = 7.3 Hz; 3H; H_3_-18); 0.93 (dq; *J* = 13.7. 7.3 Hz; 1H; H_x_-19); 1.43 (dq; *J* = 13.7. 7.3 Hz; 1H; H_y_-19); 2.11–2.19 (m; 2H; H_2_-6); 2.50–2.56 (m; 1H; H_x_-5); 2.57 (s; 3H; N(1)-CH_3_); 2.60 (s; 1H; H-21); 2.77 (ddd; *J* = 16.1. 2.5. 1.5 Hz; 1H; H_x_-3); 3.22–3.28 (m; 1H; H_y_-5); 3.37 (ddd; *J* = 16.1. 4.9. 1.2 Hz; 1H; H_y_-3); 3.38 (t; *J* = 2.4 Hz; 1H; H-3′); 3.44 (s; 1H; H-2); 3.68 (s; 1H; H-17); 3.69 (s; 6H; C(16)-COOCH_3_. C(11)-OCH_3_); 4.20–4.27 (m; 2H; H_2_-1′); 5.46 (ddd; *J* = 10.2. 2.5. 1.2 Hz; 1H; H-15); 5.78 (ddd; *J* = 10.2. 4.9. 1.5 Hz; 1H; H-14); 6.16 (d; *J* = 2.3 Hz; 1H; H-12); 6.26 (d; *J* = 8.2. 2.3 Hz; 1H; H-10); 7.02 (d; *J* = 8.2 Hz; 1H; H-9); 8.47 (s; 1H; C(16)-OH).

^13^C NMR (125.7 MHz; DMSO-*d*_6_) *δ* (ppm) 7.7 (C-18); 32.1 (C-19); 38.4 (N(1)-CH_3_); 43.2 (C-20); 44.0 (C-6); 50.5 (C-3); 51.1 (C-5); 51.6 (C(16)-COOCH_3_); 51.9 (C-7); 55.0 (C(11)-OCH_3_); 60.7 (C-1′); 66.6 (C-21); 76.5 (C-3′); 79.9 (C-16); 81.1 (C-2′); 83.4 (C-2); 84.3 (C-17); 95.3 (C-12); 104.3 (C-10); 122.9 (C-9); 123.0 (C-14); 125.5 (C-8); 131.2 (C-15); 153.4 (C-13); 160.4 (C-11); 172.6 (C(16)-COOCH_3_).

ESI-HRMS: M + H = 453.23785 (delta = −1.2ppm; C_26_H_33_O_5_N_2_). HR-ESI-MS-MS (CID = 35%; rel. int. %): 435(21); 397(45); 393(100); 188(32).

### 3.13. The Click Reaction of 17-Propargylvindoline (**20**) and 4-Fluorobenzyl-Azide (**18**)

205 mg (0.45 mmol) of 17-propargylvindoline (**20**), 72 mg (0.48 mmol) of 4-fluorobenzyl-azide, 24 mg (0.09 mmol) of triphenylphosphine and 9 mg (0.05 mmol) of copper (I) iodide were dissolved in 10 mL of dry toluene, then 0 24 mL (1.4 mmol) of DIPEA was added, and the reaction mixture was stirred at reflux for 5 h. Then, 60 mL of toluene was added, and the mixture was washed with 75 mL of distilled water, finally the aqueous phase was washed with another 25 mL of toluene. The organic phase was dried over MgSO_4_, then the solvent evaporated under reduced pressure. After preparative TLC (dichloromethane-methanol = 30:1) 126 mg (46%) of a yellow crystalline product (**21**) was obtained. Mp 92–95 °C. TLC (dichloromethane-methanol = 30:1); R*_f_* = 0,24. IR (KBr): 1085, 1224; 1245; 1502; 1736; 2837; 2946 cm^−1^.

^1^H NMR (799.7 MHz; DMSO-*d*_6_) *δ* (ppm) 0.50 (t; *J* = 7.3 Hz; 3H; H_3_-18); 0.98 (dq; *J* = 13.7. 7.3 Hz; 1H; H_x_-19); 1.38 (dq; *J* = 13.7. 7.3 Hz; 1H; H_y_-19); 2.13–2.17 (m; 2H; H_2_-6); 2.50–2.54 (m; 1H; H_x_-5); 2.59 (s; 3H; N(1)-CH_3_); 2.61 (s; 1H; H-21); 2.74–2.78 (m; 1H; H_x_-3); 3.21–3.25 (m; 1H; H_y_-5); 3.32–3.36 (m; 1H; H_y_-3); 3.44 (s; 1H; H-2); 3.66 (s; 3H; C(16)-COOCH_3_); 3.70 (s; 3H; C(11)-OCH_3_); 3.85 (s; 1H; H-17); 4.62.–4.68 (AB; *J* = 11.2 Hz; 2H; H_2_-1′); 5.42–5.45 (m; 1H; H-15); 5.56 (~s; 2H; H_2_-7′); 5.73 (ddd; *J* = 10.2. 5.0. 1.5 Hz; 1H; H-14); 6.17 (d; *J* = 2.3 Hz; 1H; H-12); 6.27 (dd; *J* = 8.2. 2.3 Hz; 1H; H-10); 7.02 (d; *J* = 8.2 Hz; 1H; H-9); 7.18–7.21 (m; 2H; H-10′. H-12′); 7.37–7.40 (m; 2H; H-9′. H-13′); 7.97 (s; 1H; H-6′); 8.43 (br s; 1H; C(16)-OH).

^13^C NMR (201.1 MHz; DMSO-*d*_6_) *δ* (ppm) 7.7 (C-18); 32.5 (C-19); 38.4 (N(1)-CH_3_); 43.4 (C-20); 44.1 (C-6); 50.5 (C-3); 51.0 (C-5); 51.5 (C(16)-COOCH_3_); 51.7 (C-7′); 51.9 (C-7); 54.9 (C(11)-OCH_3_); 66.56 (C-1′); 66.60 (C-21); 80.0 (C-16); 83.4 (C-2); 83.6 (C-17); 95.3 (C-12); 104.2 (C-10); 115.5 (d; ^2^*J*_FC_ = 21.6 Hz; C-10′. C-12′); 122.9 (C-9); 123.0 (C-14); 123.6 (C-6′); 125.6 (C-8); 130.2 (d; ^3^*J*_FC_ = 8.4 Hz; C-9′. C-13′); 131.1 (C-15); 132.3 (d; ^4^*J*_FC_ = 3.0 Hz; C-8′); 144.7 (C-2′); 153.4 (C-13); 160.4 (C-11); 161.7 (d; ^1^*J*_FC_ = 244.4 Hz; C-11′); 172.6 (C(16)-COOCH_3_).

^15^N NMR (81.0 MHz; DMSO-*d*_6_) *δ* (ppm) −322.7 (N-4); −314.1 (N-1); −131.5 (N-5′); −28.6 (N-3′); -20.5 (N-4′).

ESI-HRMS: M + H = 604.29147 (delta = −2.5ppm; C_33_H_39_O_5_N_5_F). HR-ESI-MS-MS (CID = 35%; rel. int. %): 586(18); 554(19); 544(82); 484(9); 415(100); 397(43); 377(27); 365(17); 355(28); 188(22).

### 3.14. N-Alkylation of Morpholine with 17-(4-Bromobutanoyloxy)vindoline (**22**)

17-(4-Bromobutanoyloxy)vindoline (**22**) [22,23] (120 mg, 0.21 mmol) was dissolved in 6 mL of anhydrous DCM, and the stirring was started under argon. Then, 0,04 mL (0.43 mmol) of morpholine was added. The reaction mixture was stirred at room temperature for 2 h, then at reflux for 11 h. The mixture was then allowed to cool to room temperature. After the addition of 4 mL of DCM, the mixture was extracted with 6 mL of 10% Na_2_CO_3_ solution. The aqueous phase was washed with 6 mL of DCM, then the organic phase was washed with 6 mL of distilled water. The organic phase was dried over MgSO_4_, then the solvent evaporated under reduced pressure. After preparative TLC (dichloromethane-methanol = 15:1) 90 mg (74%) of a yellow oleic product (**23**) was obtained. TLC (dichloromethane-methanol = 20:1); R*_f_* = 0.27. IR (KBr): 2954, 2808, 1735, 1613, 1500, 1223, 1115, 1024 cm^−1^.

^1^H NMR (499.9 MHz; CDCl_3_) *δ* (ppm) 0.48 (t; *J* = 7.4 Hz; 3H; H_3_-18); 1.14 (dq; *J* = 14.4. 7.4 Hz; 1H; H_x_-19); 1.64 (dq; *J* = 14.4. 7.4 Hz; 1H; H_y_-19); 1.78–1.84 (m; 2H; H_2_-3′); 2.27–2.41 (m; 6H; H_2_-6. H_2_-4′. H_2_-2′); 2.41–2.48 (br m; 4H; H_2_-6′. H_2_-10′); 2.49–2.55 (m; 1H; H_x_-5); 2.66 (s; 1H; H-21); 2.67 (s; 3H; N(1)-C*H_3_*); 2.82 (ddd; *J* = 16.0. 2.6. 1.6 Hz; 1H; H_x_-3); 3.39–3.45 (m; 1H; H_y_-5); 3.49 (ddd; *J* = 16.0. 4.9. 1.3 Hz; 1H; H_y_-3); 3.67–3.73 (br m; 4H; H_2_-7′. H_2_-9′); 3.74 (s; 1H; H-2); 3.779 (s; 3H). 3.780 (s; 3H): C(16)-COOC*H_3_*. C(11)-OC*H_3_*; 5.23 (ddd; *J* = 10.2. 2.6. 1.3 Hz; 1H; H-15); 5.46 (s; 1H; H-17); 5.84 (ddd; *J* = 10.2. 4.9. 1.6 Hz; 1H; H-14); 6.07 (d; *J* = 2.3 Hz; 1H; H-12); 6.30 (dd; *J* = 8.2. 2.3 Hz; 1H; H-10); 6.89 (d; *J* = 8.2 Hz; 1H; H-9); 9.52 (br; 1H; C(16)-O*H*).

^13^C NMR (125.7 MHz; CDCl_3_) *δ* (ppm) 7.7 (C-18); 21.7 (br; C-3′); 30.8 (C-19); 31.8 (C-2′); 38.3 (N(1)-*C*H_3_); 42.9 (C-20); 44.1 (C-6); 51.1 (C-3); 52.0 (C-5); 52.2 (C(16)-COO*C*H_3_); 52.8 (C-7); 53.5 (br; C-6′. C-10′); 55.4 (C(11)-O*C*H_3_); 57.9 (C-4′); 66.9 (br; C-7′. C-9′); 67.1 (C-21); 76.3 (C-17); 79.6 (C-16); 83.4 (C-2); 95.8 (C-12); 104.6 (C-10); 122.7 (C-9); 124.1 (C-14); 125.1 (C-8); 130.6 (C-15); 153.7 (C-13); 161.2 (C-11); 171.9 (C(16)-*C*OOCH_3_); 173.2 (C-1′).

ESI-HRMS: M + H = 570.31692 (delta = −0.8 ppm; C_31_H_44_O_7_N_3_). HR-ESI-MS-MS (CID = 35%; rel. int. %): 552(100); 415(2); 397(25); 188(1).

### 3.15. N-Alkylation of Piperazine with 17-(4-Bromobutanoyloxy)vindoline (**22**)

17-(4-Bromobutanoyloxy)vindoline (**22**) [22,23] (140 mg, 0.25 mmol) was dissolved in 6 mL of anhydrous DCM, and the stirring was started under argon. Then, 43 mg (0.50 mmol) of piperazine was added. The reaction mixture was stirred at reflux for 7 h. The mixture was then allowed to cool to room temperature, and after the addition of a few DCM, the mixture was extracted with 6 mL of 10% Na_2_CO_3_ solution. The aqueous phase was washed with 6 mL of DCM, then the organic phase was washed with 6 mL of distilled water. The organic phase was dried over MgSO_4_, then the solvent evaporated under reduced pressure. After preparative TLC (dichloromethane-methanol = 15:1) 47 mg (36%) of a pale yellow crystalline product (**24**) was obtained. Mp 118–124 °C. TLC (dichloromethane-methanol = 15:1); R*_f_* = 0,05. IR (KBr): 2947, 2808, 1740, 1616 1502, 1246, 1170 cm^−1^.

The molecule is symmetric, it has two equivalent halves. The NMR assignment below applies to the other half of the molecule as well.

^1^H NMR (499.9 MHz; DMSO-*d*_6_) *δ* (ppm) 0.42 (t; *J* = 7.4 Hz; 6H; H_3_-18); 0.95 (dq; *J* = 13.9. 7.4 Hz; 2H; H_x_-19); 1.48 (dq; *J* = 13.9. 7.4 Hz; 2H; H_y_-19); 1.54–1.68 (m; 4H; H_2_-3′); 2.16–2.28 (m; 12H; H_2_-6. H_2_-2′. H_2_-4′); 2.22–2.43 (br; 8H; H_2_-6′. H_2_-7′); 2.53–2.60 (m; 8H; N(1)-C*H_3_*. H_x_-5); 2.64 (s; 2H; H-21); 2.79 (br d; *J* = 16.4 Hz; 2H; H_x_-3); 3.25–3.31 (m; 2H; H_y_-5); 3.41 (br dd; *J* = 16.4. 4.8 Hz; 2H; H_y_-3); 3.54 (s; 2H; H-2); 3.63 (s; 6H; C(16)-COOC*H_3_*); 3.70 (s; 6H; C(11)-OC*H_3_*); 5.09 (br d; *J* = 10.2 Hz; 2H; H-15); 5.19 (s; 2H; H-17); 5.81 (ddd; *J* = 10.2. 4.8. 1.2 Hz; 2H; H-14); 6.19 (d; *J* = 2.2 Hz; 2H; H-12); 6.28 (dd; *J* = 8.2. 2.2 Hz; 2H; H-10); 7.05 (d; *J* = 8.2 Hz; 2H; H-9); 8.75 (s; 2H; C(16)-O*H*).

^13^C NMR (125.7 MHz; DMSO-*d*_6_) *δ* (ppm) 7.5 (C-18); 21.5 (C-3′); 30.3 (C-19); 31.3 (C-2′); 38.0 (N(1)-*C*H_3_); 42.4 (C-20); 43.6 (C-6); 50.4 (C-3); 51.1 (C-5); 51.6 (C(16)-COO*C*H_3_); 52.0 (C-7); 52.6 (C-6′. C-7′); 55.0 (C(11)-O*C*H_3_); 56.6 (C-4′); 66.1 (C-21); 75.7 (C-17); 78.6 (C-16); 82.8 (C-2); 95.4 (C-12); 104.5 (C-10); 123.0 (C-9); 124.3 (C-14); 125.3 (C-8); 129.9 (C-15); 153.4 (C-13); 160.4 (C-11); 171.6 (C(16)-*C*OOCH_3_); 172.4 (C-1′).

ESI-HRMS: M + H = 1051.57418 (delta = −0.8 ppm; C_58_H_79_O_12_N_6_). HR-ESI-MS-MS (CID = 35%; rel. int. %): 1034(100); 1016(10); 655(2); 637(22); 619(11); 397(12).

### 3.16. N-Alkylation of N-Methylpiperazine with 17-(4-Bromobutanoyloxy)vindoline (**22**)

17-(4-Bromobutanoyloxy)vindoline (**22**) [22,23] (180 mg, 0.32 mmol) was dissolved in 8 mL of anhydrous DCM, and the stirring was started under argon. Then, 0,072 mL (0.64 mmol) of *N*-methylpiperazine was added. The reaction mixture was stirred at reflux for 12 h. The mixture was then allowed to cool to room temperature, and after the addition of a few DCM, the mixture was extracted with 6 mL of 10% Na_2_CO_3_ solution. The aqueous phase was washed with 6 mL of DCM, then the organic phase was washed with 6 mL of distilled water. The organic phase was dried over MgSO_4_, then the solvent evaporated under reduced pressure. After preparative TLC (dichloromethane-methanol = 15:1) 80 mg (43%) of a white crystalline product (**25**) was obtained. Mp 65–70 °C. TLC (dichloromethane-methanol = 20:1); R*_f_* = 0.06. IR (KBr): 2937, 2794, 1740, 1616, 1502, 1244, 1166 cm^−1^.

^1^H NMR (499.9 MHz; DMSO-*d*_6_) *δ* (ppm) 0.42 (t; *J* = 7.4 Hz; 3H; H_3_-18); 0.95 (dq; *J* = 14.0. 7.4 Hz; 1H; H_x_-19); 1.49 (dq; *J* = 14.0. 7.4 Hz; 1H; H_y_-19); 1.55–1.68 (m; 2H; H_2_-3′); 2.13 (s; 3H; H_3_-11′); 2.16–2.28 (m; 6H; H_2_-6. H_2_-2′. H_2_-4′); 2.17–2.44 (br; 8H; H_2_-7′. H_2_-9′. H_2_-6′. H_2_-10′); 2.53–2.61 (m; 4H; N(1)-C*H_3_*. H_x_-5); 2.66 (s; 1H; H-21); 2.79 (br d; *J* = 16.3 Hz; 1H; H_x_-3); 3.25–3.31 (m; 1H; H_y_-5); 3.41 (br dd; *J* = 16.3. 4.9 Hz; 1H; H_y_-3); 3.54 (s; 1H; H-2); 3.64 (s; 3H; C(16)-COOC*H_3_*); 3.71 (s; 3H; C(11)-OC*H_3_*); 5.10 (br d; *J* = 10.2 Hz; 1H; H-15); 5.19 (s; 1H; H-17); 5.81 (ddd; *J* = 10.2. 4.9. 1.2 Hz; 1H; H-14); 6.19 (d; *J* = 2.2 Hz; 1H; H-12); 6.28 (dd; *J* = 8.2. 2.2 Hz; 1H; H-10); 7.05 (d; *J* = 8.2 Hz; 1H; H-9); 8.76 (s; 1H; C(16)-O*H*).

^13^C NMR (125.7 MHz; DMSO-*d*_6_) *δ* (ppm) 7.5 (C-18); 21.5 (C-3′); 30.3 (C-19); 31.2 (C-2′); 38.0 (N(1)-*C*H_3_); 42.4 (C-20); 43.6 (C-6); 45.6 (C-11′); 50.4 (C-3); 51.1 (C-5); 51.6 (C(16)-COO*C*H_3_); 52.0 (C-7); 52.5 (C-6′. C-10′); 54.6 (C-7′. C-9′); 55.0 (C(11)-O*C*H_3_); 56.6 (C-4′); 66.1 (C-21); 75.7 (C-17); 78.7 (C-16); 82.8 (C-2); 95.4 (C-12); 104.5 (C-10); 123.0 (C-9); 124.3 (C-14); 125.3 (C-8); 129.9 (C-15); 153.4 (C-13); 160.5 (C-11); 171.6 (C(16)-*C*OOCH_3_); 172.4 (C-1′).

EI-HRMS: M = 582.34230 (delta = 1.9 ppm; C_32_H_46_O_6_N_4_).

### 3.17. Cell Lines and Reagents

Human breast cancer cell lines MCF-7 and MDA-MB-231, and human cervical cancer HeLa cells were purchased from ECCAC - European Collection of Cell Cultures, Salisbury, UK. SiHa cervical cancer cells were obtained from the American Type Culture Collection (ATCC), Manassas, VA, USA. Eagle’s minimum essential medium (EMEM), fetal bovine serum (FBS), penicillin–amphotericin B, non-essential amino acids, trypsin, were purchased from Lonza Group Ltd. (Basel, Switzerland) while Trypan blue solution, 3-(4,5-dimethylthiazol-2-yl)-2,5-diphenyltetrazolium bromide (MTT), and dimethyl-sulfoxide (DMSO) were from Sigma-Aldrich Ltd. (Budapest, Hungary). All the cancer cell lines were cultured in 75 cm^2^ flask (Orange Scientific, Hungary) in EMEM media according to the distributor’s instructions, supplemented with 10% fetal bovine serum, 1% non-essential amino acids and an antibiotic–antimycotic mixture. The cells were incubated in cultured conditions at 37 °C under humidified atmosphere with 5% carbon dioxide CO_2_ [33].

### 3.18. Antiproliferative Assay

The antiproliferative activity of the prepared compounds was determined by colorimetric MTT assay on a panel of human cancer cell lines isolated from breast (MCF-7 and MDA-MB-231) and cervical (HeLa and SiHa) cancers as published before [34]. Briefly, the cells were seeded in 96-well microplates with an initial cell density of 5 × 10^3^ cells per well and incubated overnight to allow cells’ attachment in the bottom of wells. Afterwards the cells were treated with mediums containing six different concentrations (0.1, 0.3, 1, 3, 10, 30 µM) of the tested compounds and incubated for 72 h. We note that, under the cell culture conditions, the highest DMSO content of the medium (0.3%) did not have any substantial effect on cell proliferation. Subsequently, the cells were incubated with 44 µL of 5 mg/mL MTT solution for 4 h. Then, the medium was discarded and the precipitated formazan crystals were solubilized in 100 µL of DMSO by gently shaking for 60 min at 37 °C. Finally, absorbance was measured at 545 nm wavelength by using a microplate reader. Antiproliferative activity was determined as a percentage of inhibition compared to the untreated cell control. IC_50_ values, representing the 50% inhibitory concentrations, were obtained from nonlinear regression using Graph Pad Prism 5.01 (Graph Pad Software; San Diego, CA, USA). All in vitro tests were carried out in two separate experiments, n = 5 each.

## 4. Conclusions

A significant antitumor effect was presumed by connecting amino acids [(l)- or (d)-tryptophan] and synthetic pharmacophores, such as a 1,2,3-triazole derivative, morpholine, piperazine and *N*-methylpiperazine to the “monomer” vindoline (**1**), a natural compound of the *Vinca* alkaloid family, which does not exhibit any antitumor effect by itself. The coupling of the key intermediates 10-aminovindoline (**5**) and 17-desacetylvindoline (**9**) with the aforementioned scaffolds resulted in new *Vinca* alkaloid derivatives. A few of these compounds (**11**, **12**, and **24**) demonstrated promising antitumor effect, and may be considered as promising leads, particularly against certain types of cervical cancer. Our results suggest that it is possible to engineer vindoline (**1**) to become a real anticancer drug by conjugating it with suitable pharmacophores.

## Figures and Tables

**Table 1:** In vitro antiproliferative activity of selected compounds against human gynecological cancer cell lines. Compounds were tested in the concentration range of 0.1–30 µM in 2 biological replicates, 5 parallel measurements each. IC_50_ values and their 95% confidence intervals (C.I.) are presented. Bold font of numbers mean stronger IC_50_ values compared to vinblastine (**3**) sulfate on SiHa cells.

IC_50_ [95% C.I.] (µM)				
COMPOUND	HeLa	SiHa	MCF-7	MDA-MB-231
**vindoline (1)**	>30	>30	>30	>30
**vinblastine (3) sulfate**	<0.1	**14.42** [12.75–16.31]	<0.1	<0.1
**17-desacetylvindoline (9)**	>30	>30	>30	>30
**7**	>30	>30	>30	>30
**8**	>30	>30	>30	>30
**11**	>30	**6.01** [5.45–6.62]	>30	8.59 [7.63–9.68]
**12**	12.03 [9.76–14.84]	**12.29** [10.29–14.68]	13.09 [10.93–15.68]	~20.41 ^a^
**14**	>30	>30	>30	>30
**15**	>30	>30	>30	>30
**19**	>30	>30	>30	>30
**21**	>30	>30	>30	>30
**23**	>30	>30	>30	>30
**24**	3.21 [2.68–3.84]	**2.85** [2.42–3.36]	3.63 [2.93–4.51]	3.52 [1.75–7.10]
**25**	9.36 [8.87–9.87]	>30	14.10 [11.36–17.50]	25.49 [22.32–29.11]

^a^ Ambiguous fitting, no confidence interval available.

## Supplementary Materials

The following are available online at https://www.mdpi.com/1420-3049/25/4/1010/s1, ^1^H and ^13^C NMR data of all the new compounds.
