# Supplementary material for: Synthesis and Cytotoxic Activity of New Vindoline Derivatives Coupled to Natural and Synthetic Pharmacophores
Source: Molecules. 2020 Feb 24;25(4):1010. doi: 10.3390/molecules25041010 (PMC7070384; doi:10.3390/molecules25041010)

molecules

SUPPLEMENTARY MATERIALS

MDPI

Article

Synthesis and Cytotoxic Activity of New Vindoline Derivatives Coupled to Natural and Synthetic Pharmacophores

András Keglevich [1,*], Leonetta Dányi [1], Alexandra Rieder [1], Dorottya Horváth [1], Áron Szigetvári [2], Miklós Dékány [2], Csaba Szántay, Jr. [2], Ahmed Dhahir Latif [3,4], Attila Hunyadi [3,5], István Zupkó [4,5], Péter Keglevich [1,*] and László Hazai [1]

[1] Department of Organic Chemistry and Technology, Budapest University of Technology and Economics, H-1111 Budapest, Gellért tér 4., Hungary; danyi.leonetta@gmail.com (L.D.); riederszandra@gmail.com (A.R.); dorkahorvath03@gmail.com (D.H); hazai@mail.bme.hu (L.H.)

[2] Spectroscopic Research Department, Gedeon Richter Plc., H-1475 Budapest 10, P. O. Box 27, Hungary; szigetvaria@richter.hu (Á.S.); m.dekany@richter.hu (M.D.); cs.szantay@richter.hu (C.S.J.)

[3] Institute of Pharmacognosy, Interdisciplinary Excellence Centre, University of Szeged, H-6720 Szeged, Eötvös u. 6., Hungary. hunyadi.a@pharm.u-szeged.hu (A.H.)

[4] Department of Pharmacodynamics and Biopharmacy, Interdisciplinary Excellence Centre, University of Szeged, H-6720 Szeged, Eötvös u. 6., Hungary. latif.ahmed@pharmacognosy.hu (A.D.L.); zupko@pharm.u-szeged.hu (I.Z.)

[5] Interdisciplinary Centre for Natural Products, University of Szeged, H-6720 Szeged, Eötvös u. 6., Hungary

* Correspondence: keglevich.andras@mail.bme.hu (A.K.); pkeglevich@mail.bme.hu (P.K.). Tel.: +36-1-463-2208

Supplementary materials - ^{1}H and ^{13}C NMR data

S.1. Compound 6

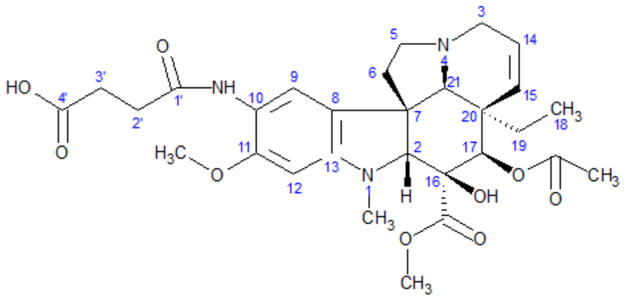

Compound **6**
^{1}H NMR (399.8 MHz; DMSO-d_6)

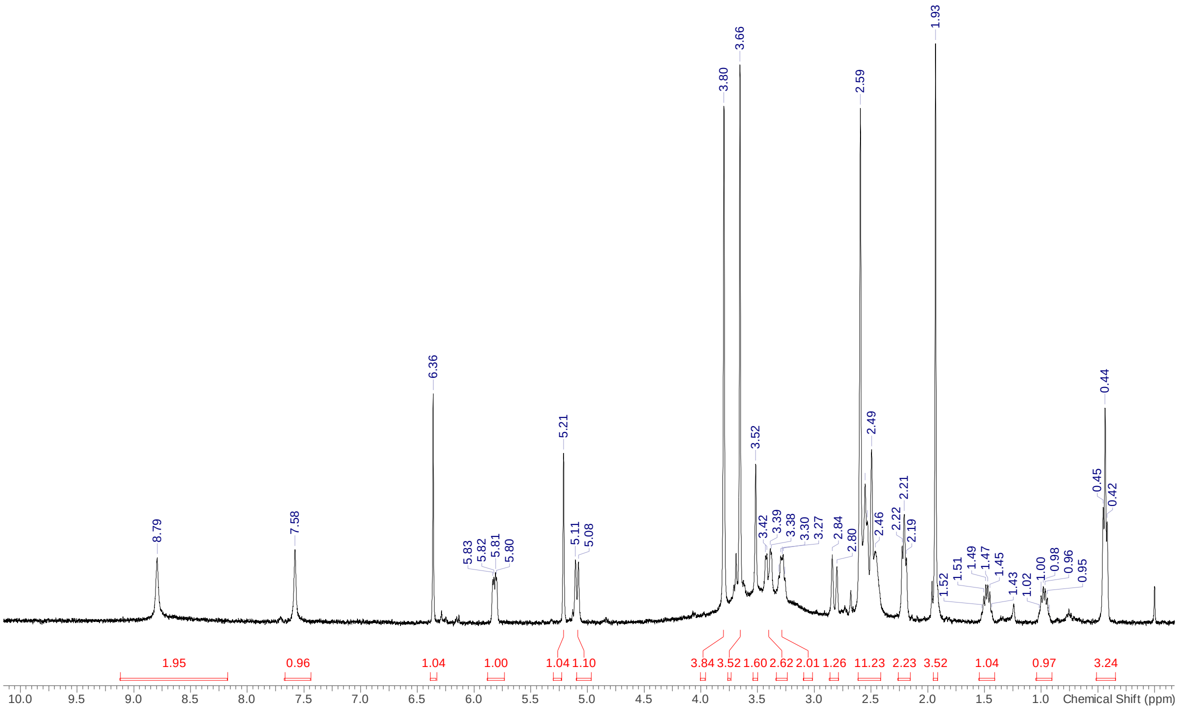

Compound **6**
^{13}C NMR (100.5 MHz; DMSO-d_6)

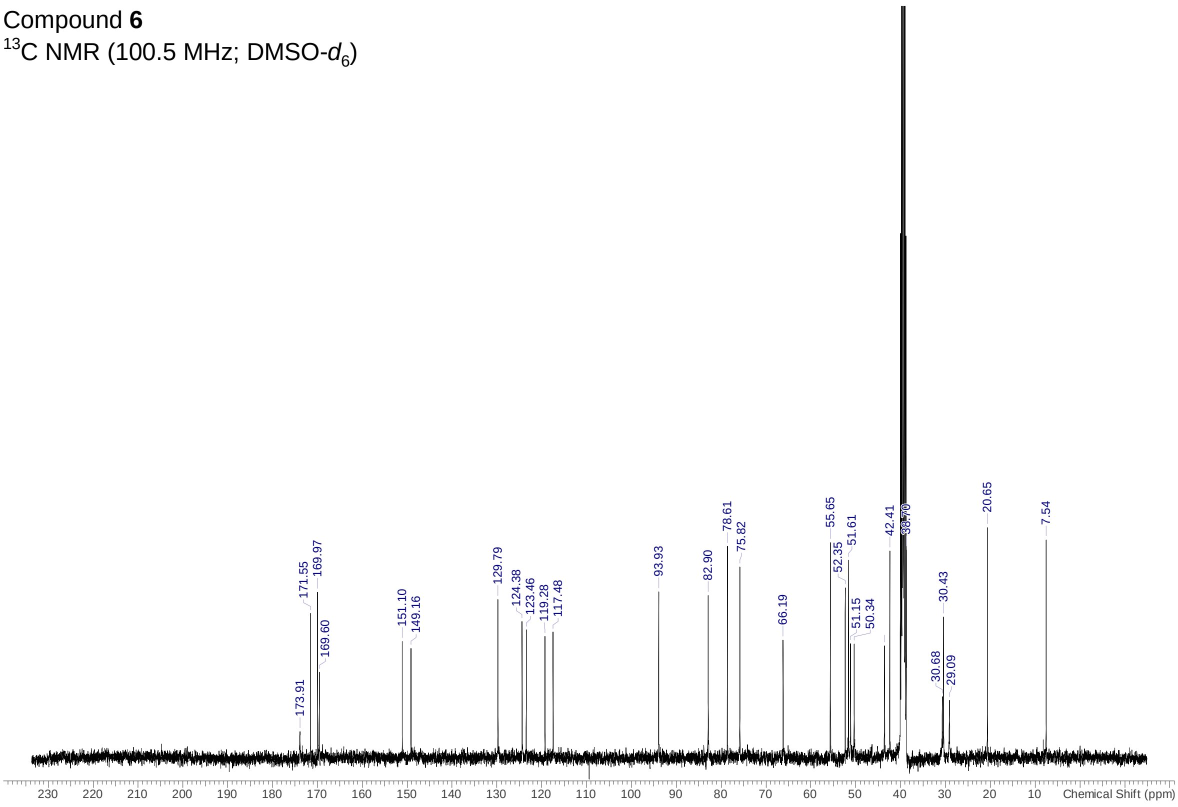

S.2. Compound 7

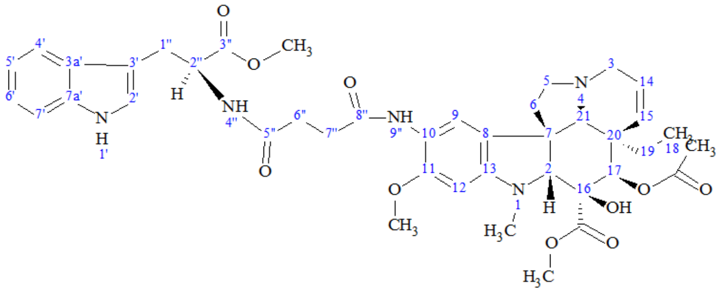

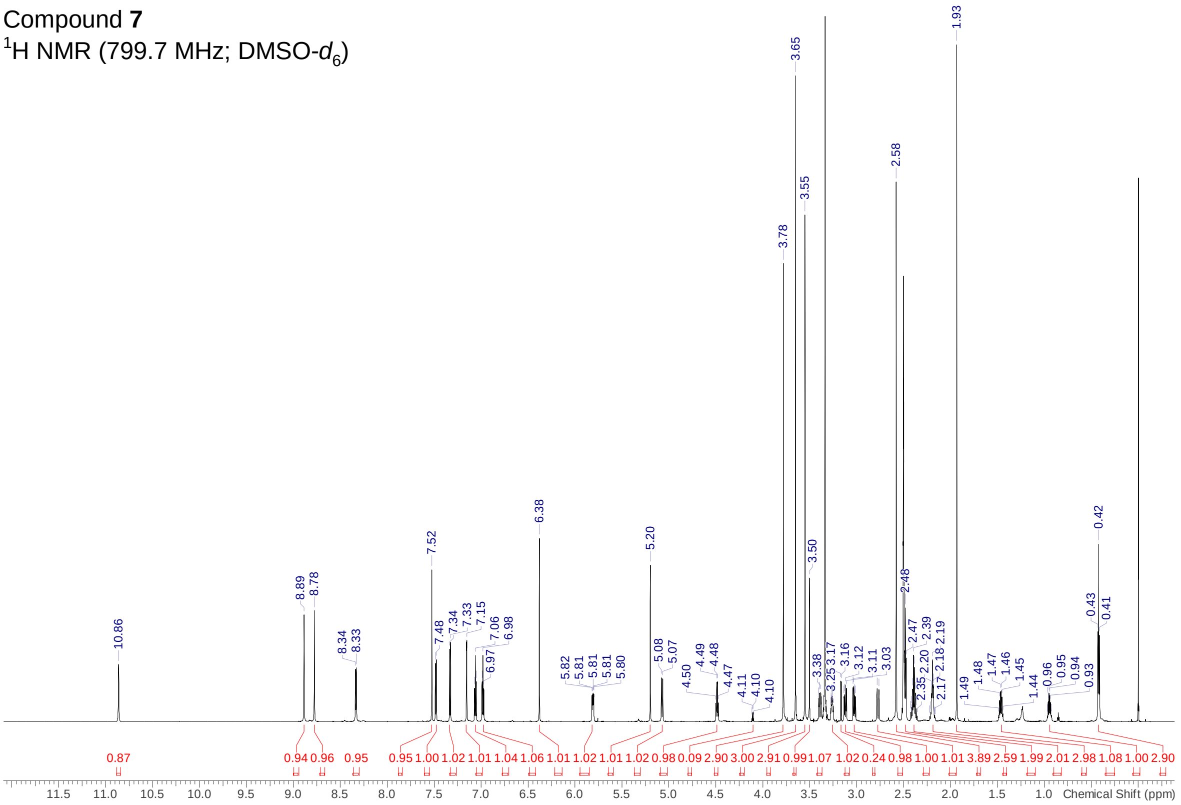

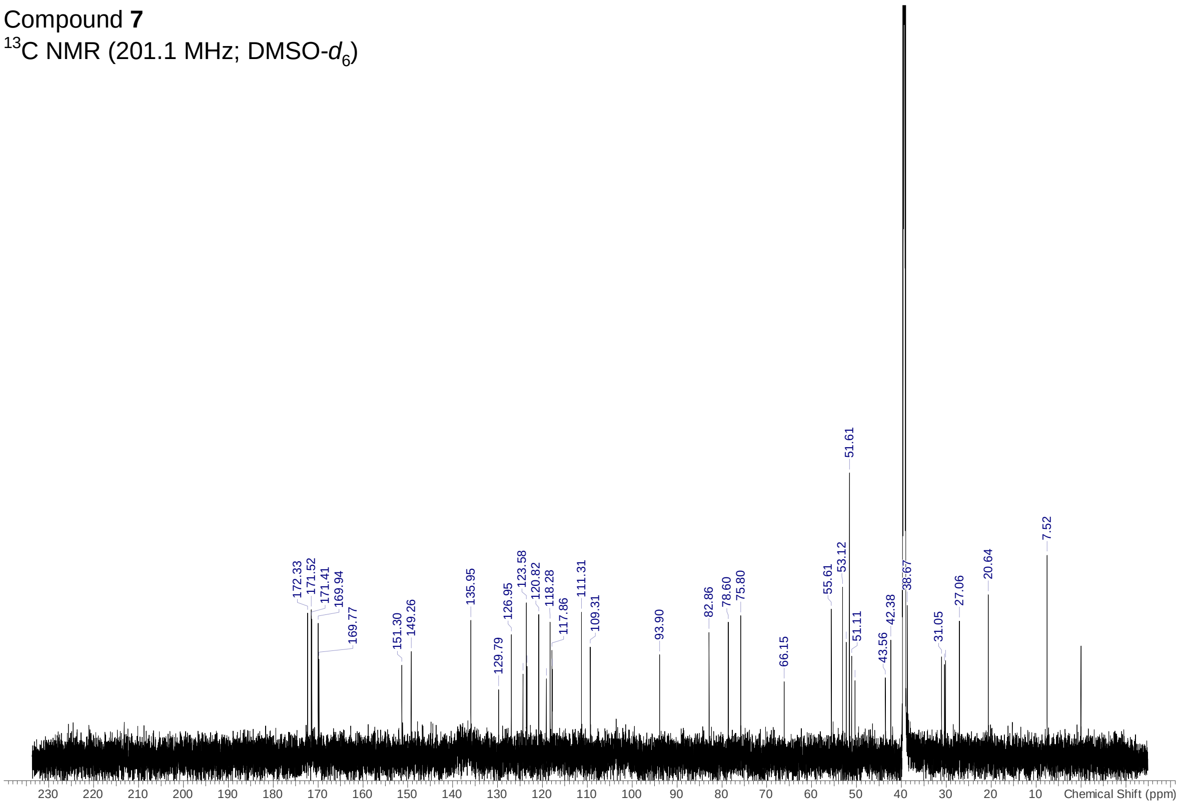

S.3. Compound 8

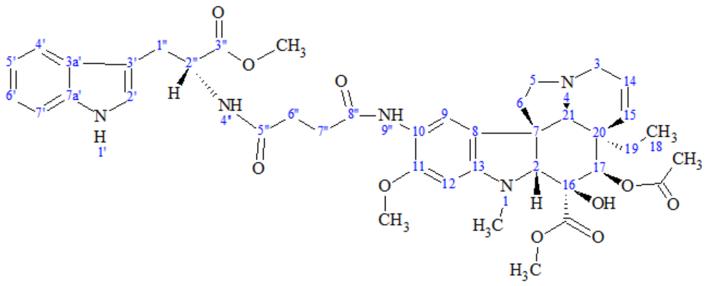

Compound **8**
^{1}H NMR (499.9 MHz; DMSO-d_6)

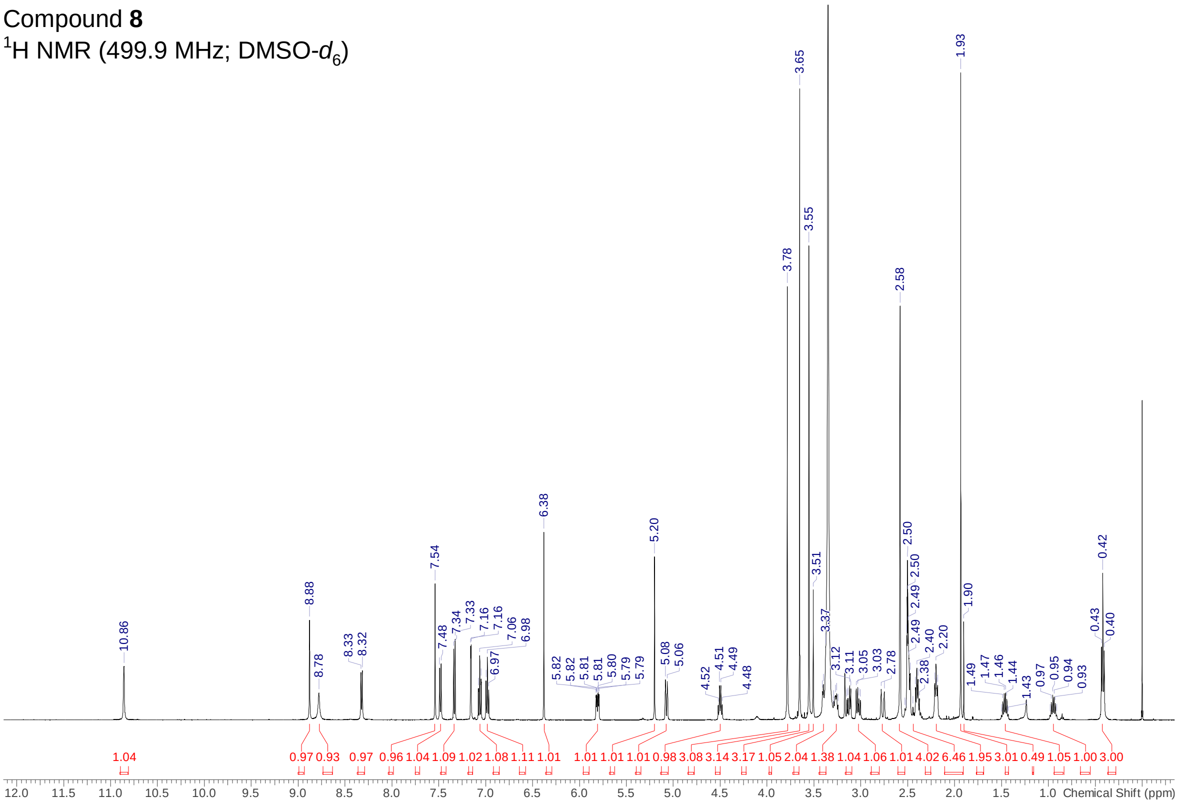

Compound **8**
^{13}C NMR (125.7 MHz; DMSO-d_6)

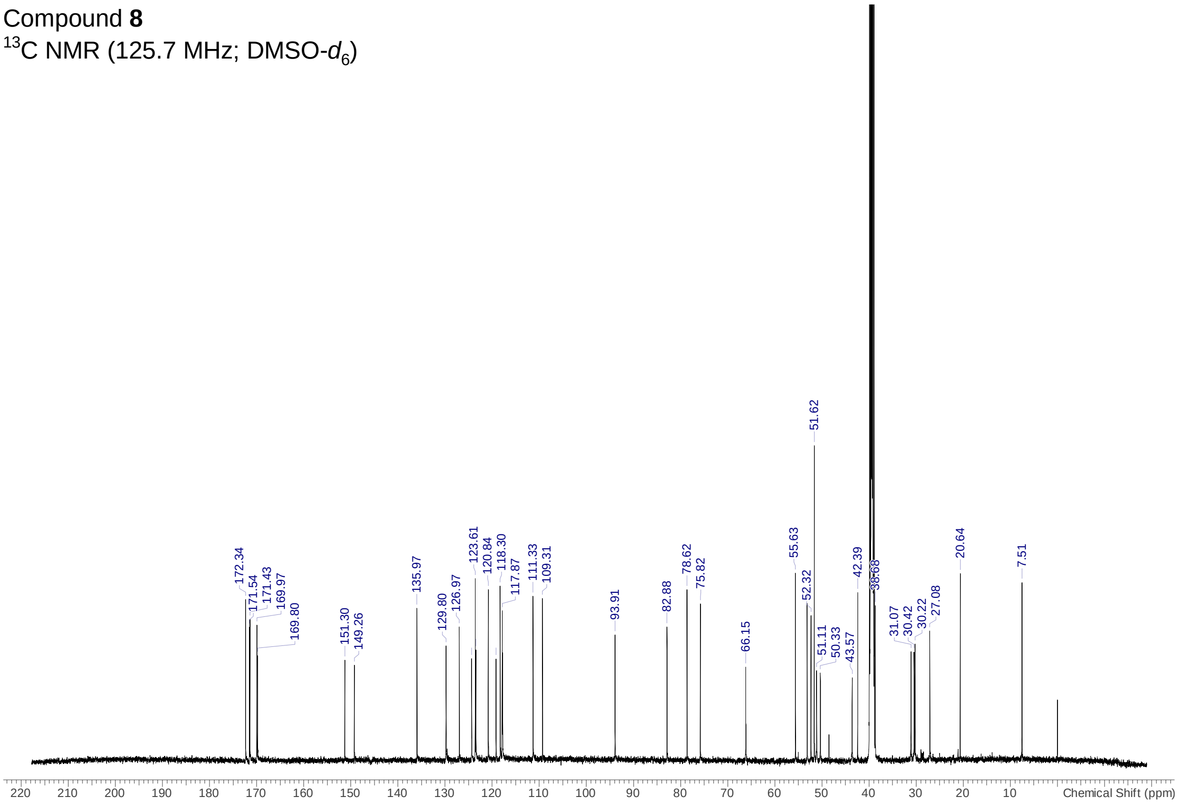

S.4. Compound 11

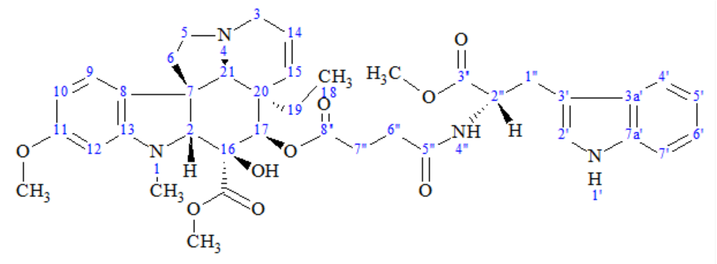

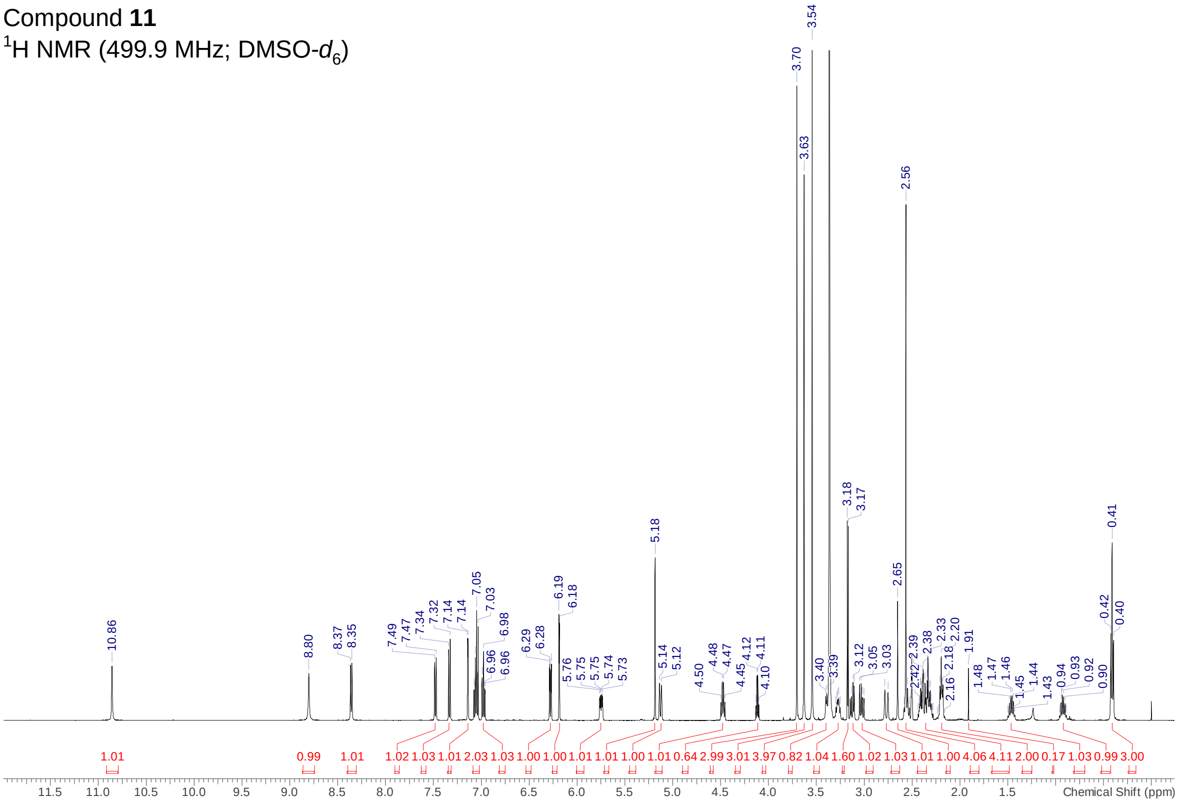

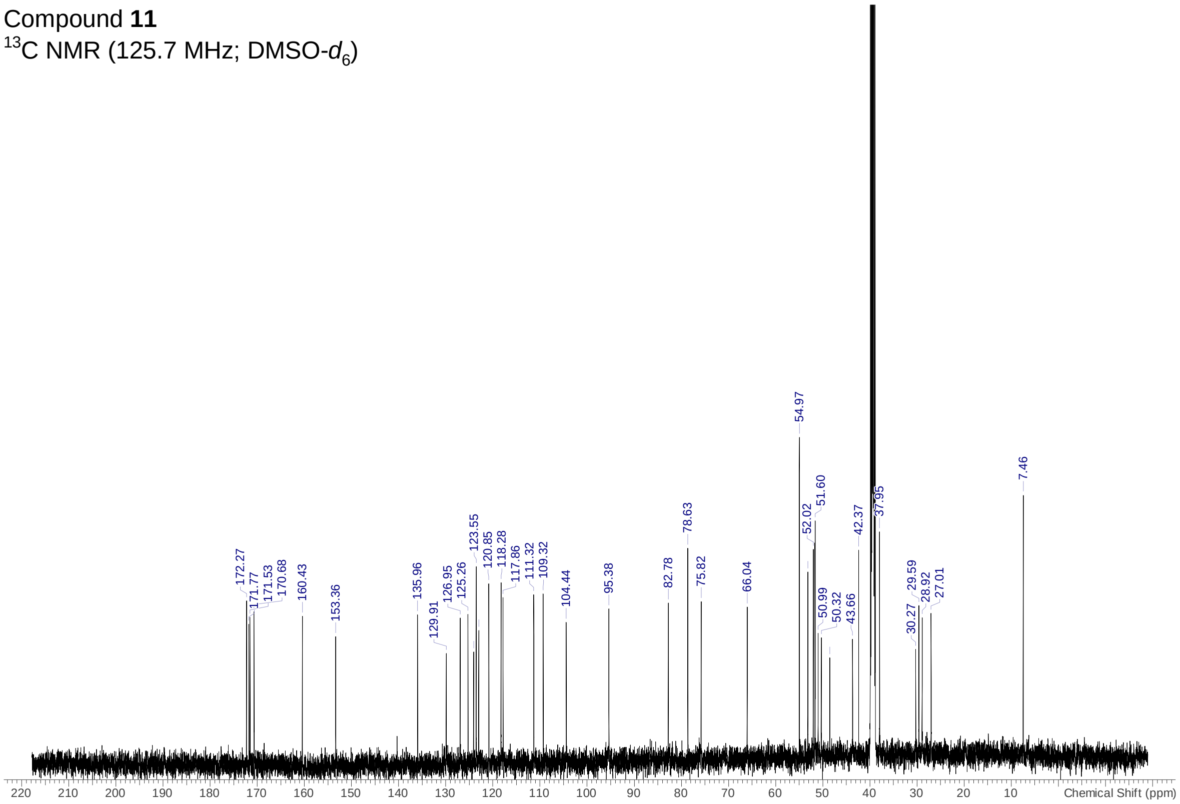

S.5. Compound 12

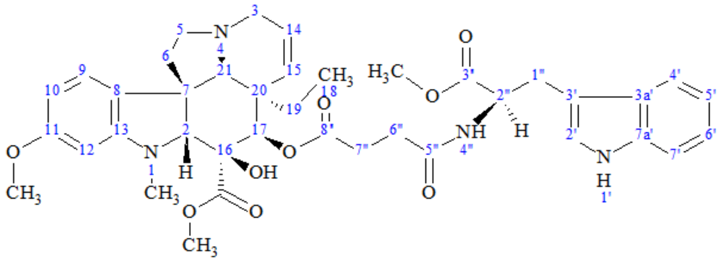

Compound **12**

^{1}H NMR (499.9 MHz; DMSO-d_6)

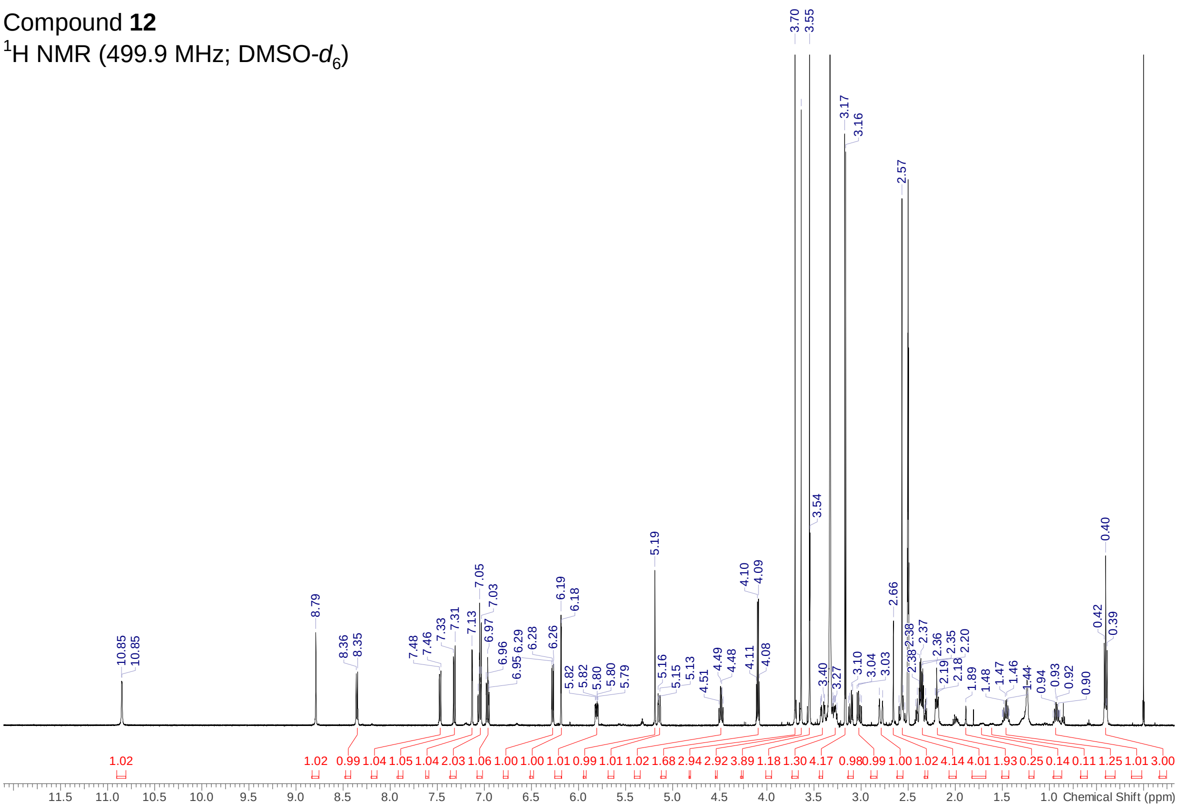

Compound **12**

^{13}C NMR (125.7 MHz; DMSO-d_6)

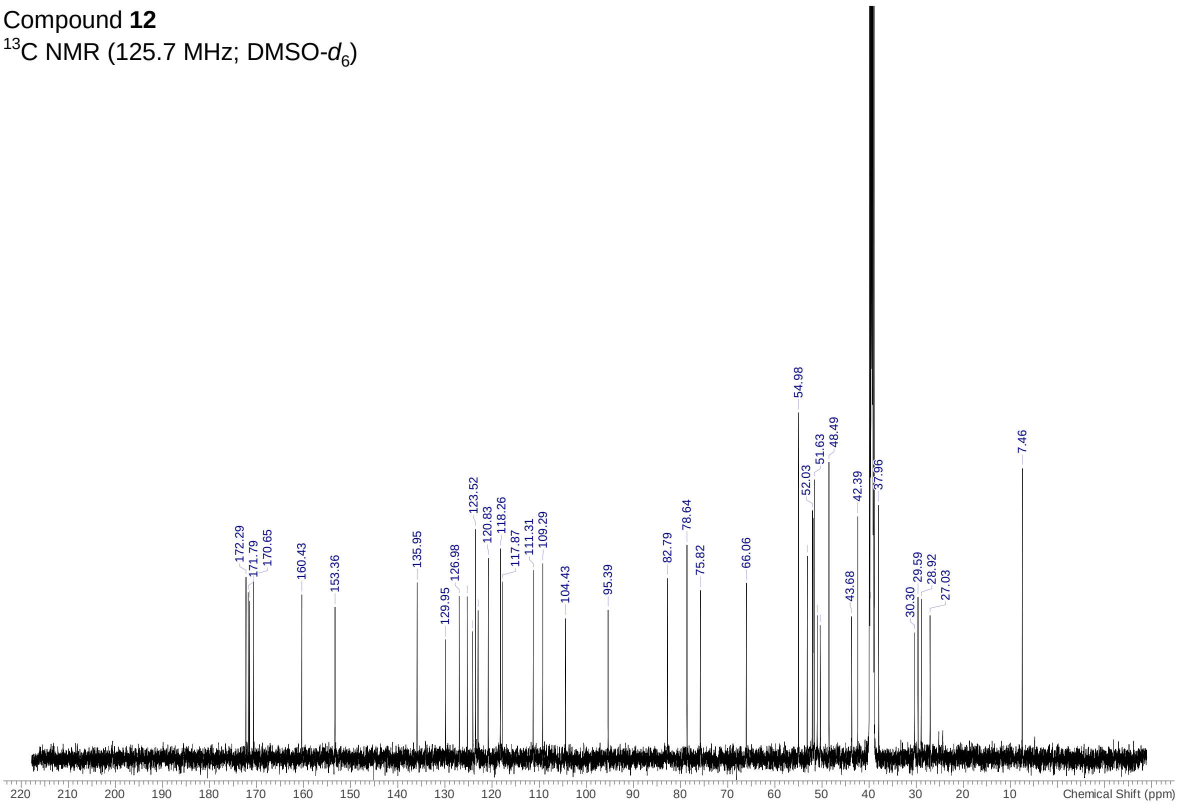

S.6. Compound 13

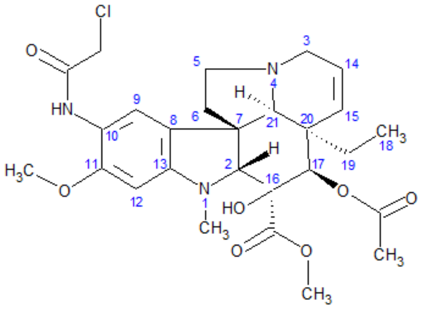

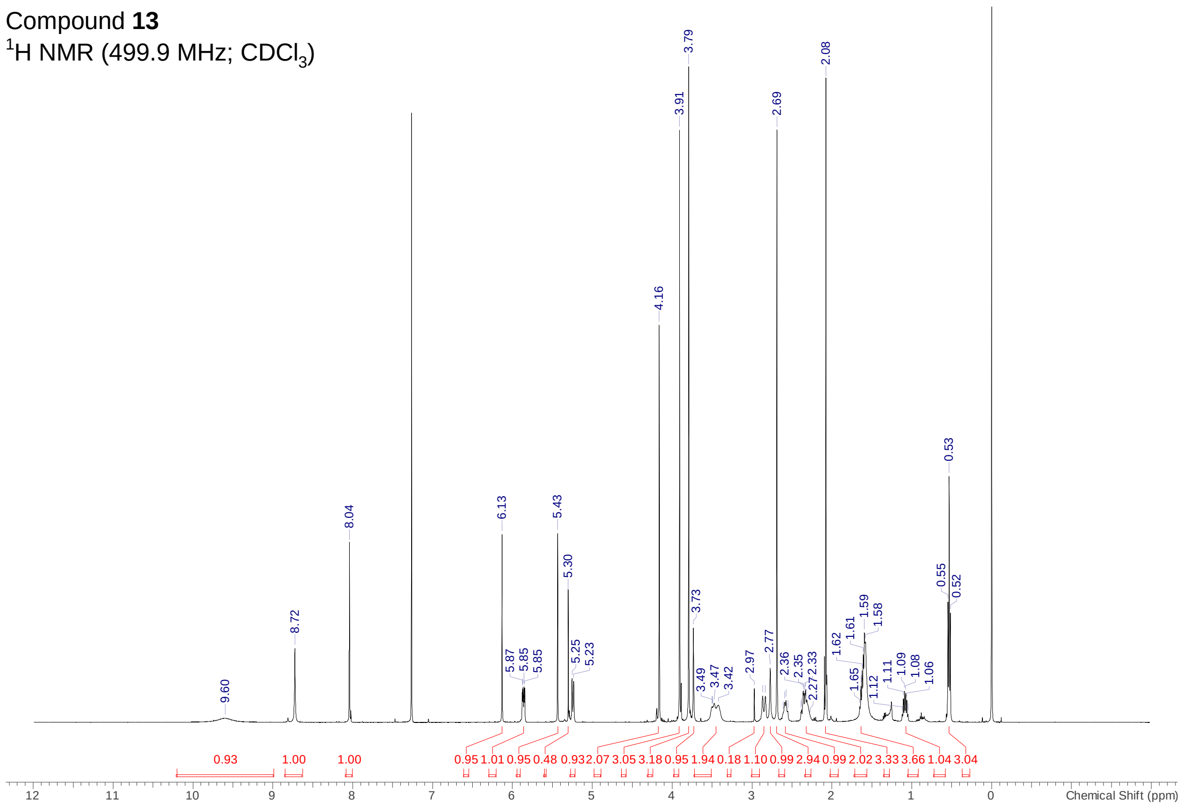

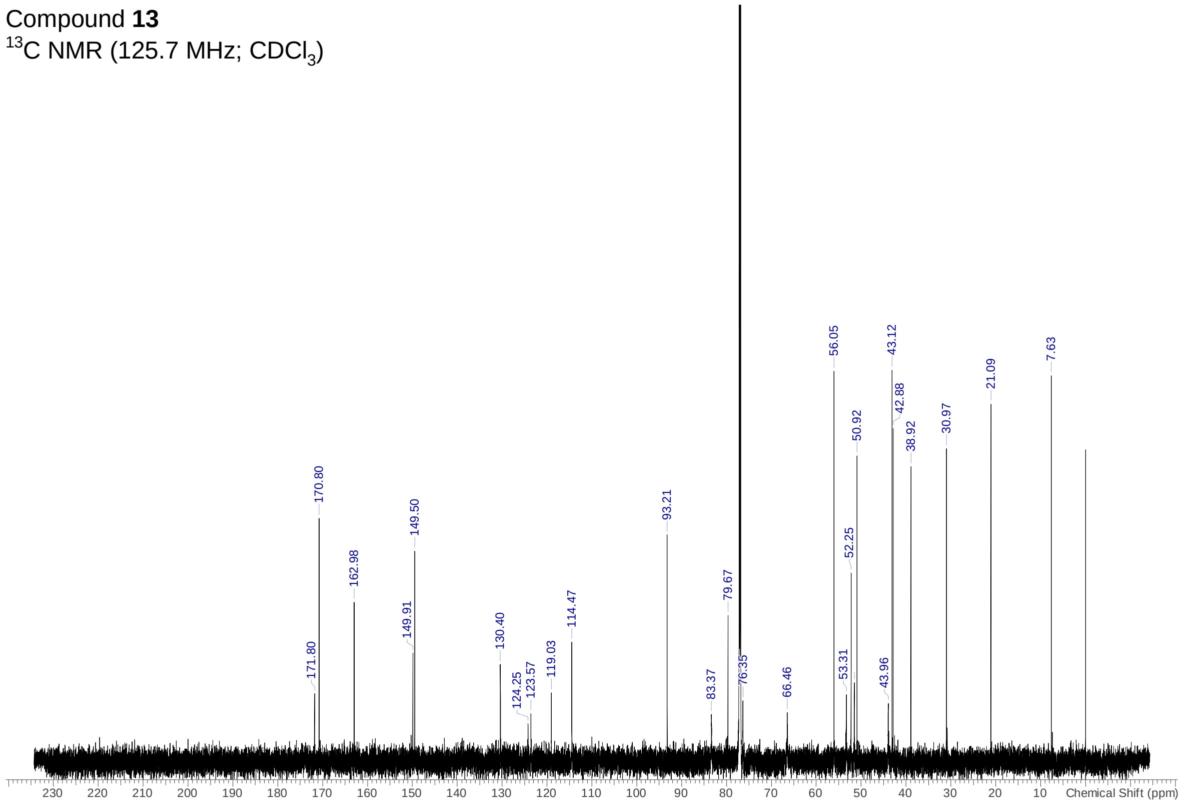

S.7. Compound 14

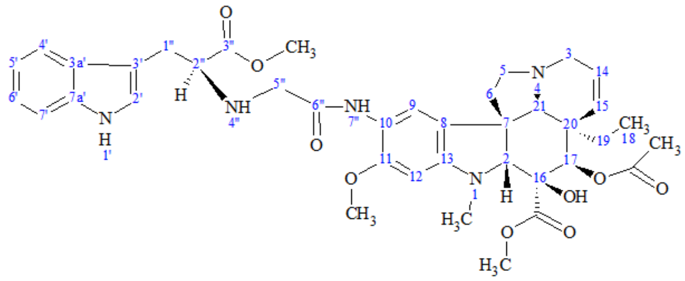

Compound **14**

^{1}H NMR (499.9 MHz; DMSO-d_6)

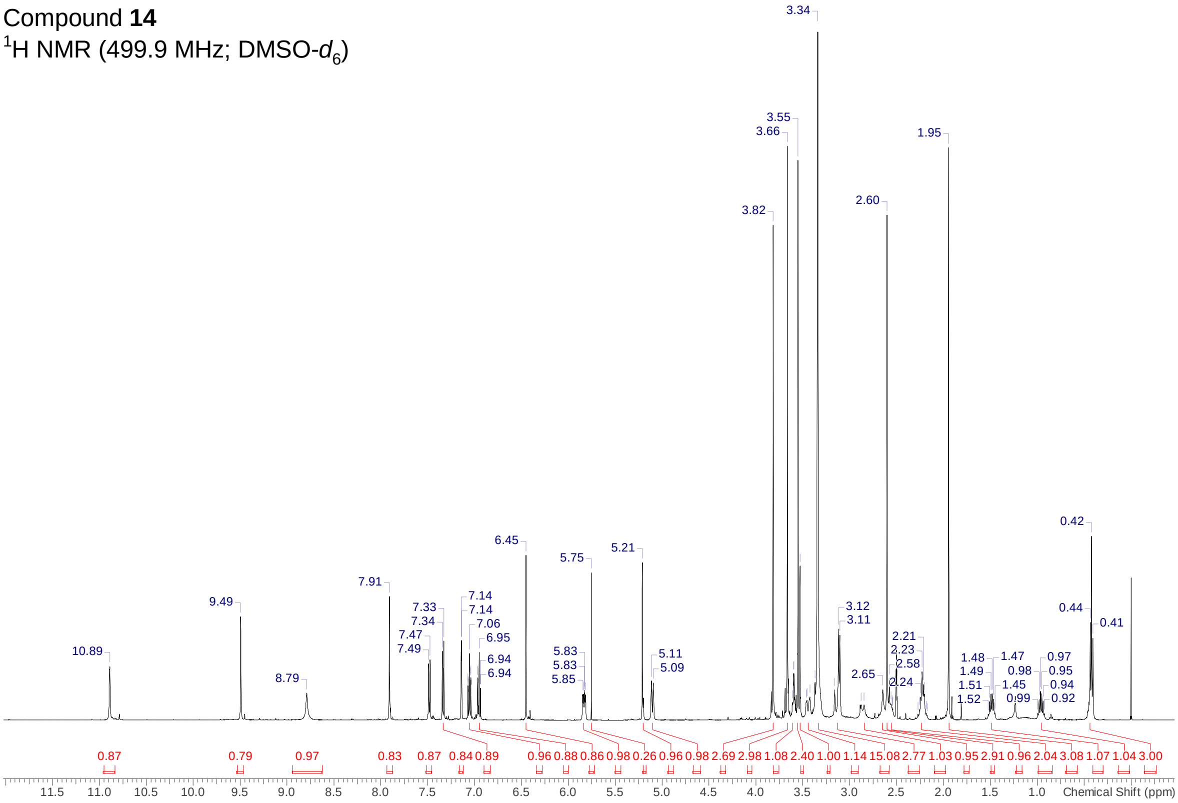

Compound **14**

^{13}C NMR (125.7 MHz; DMSO-d_6)

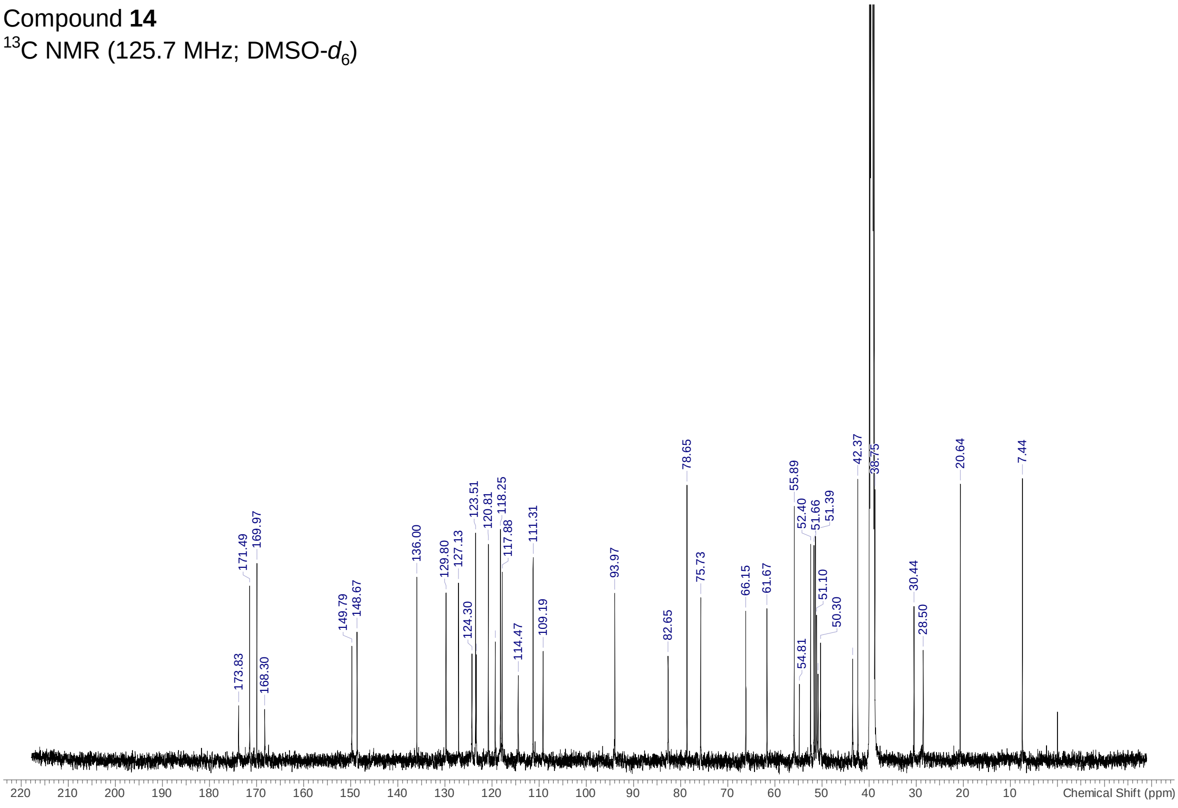

S.8. Compound 15

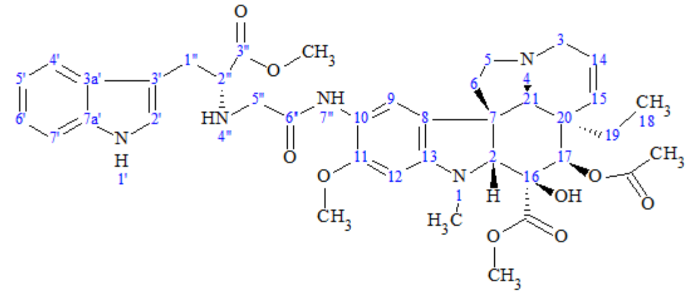

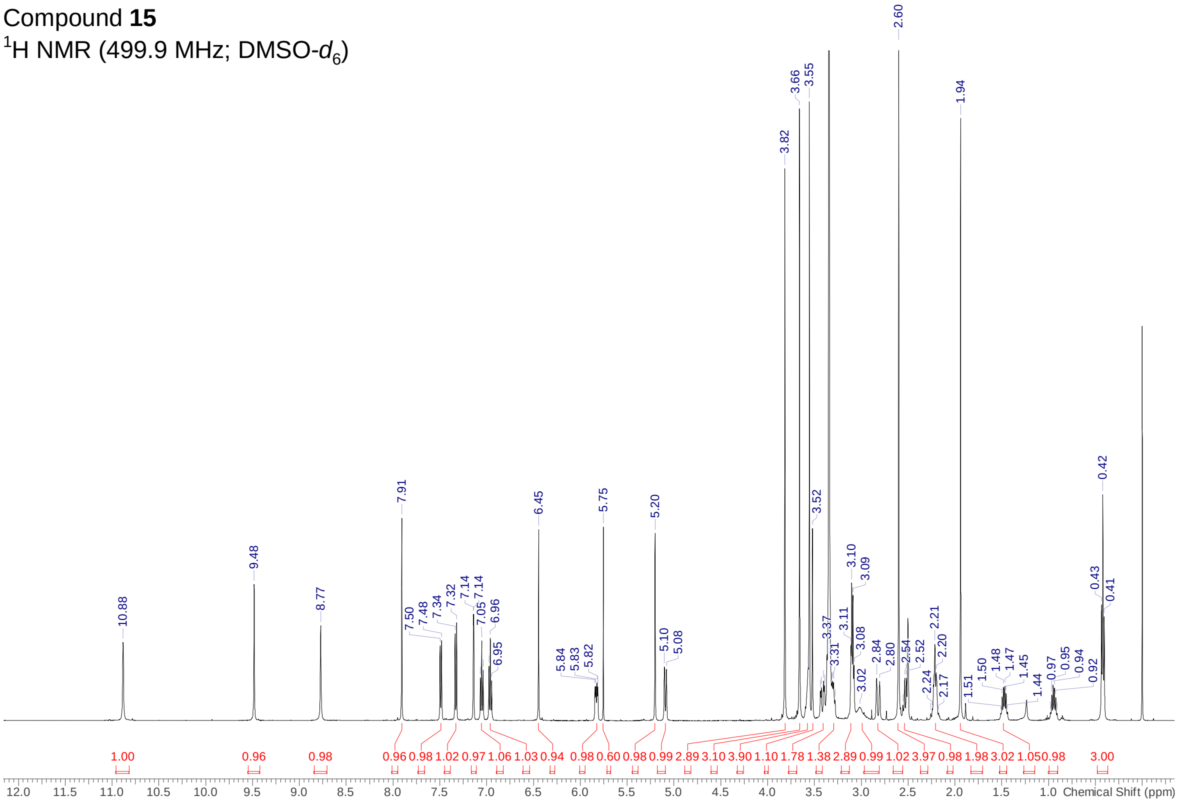

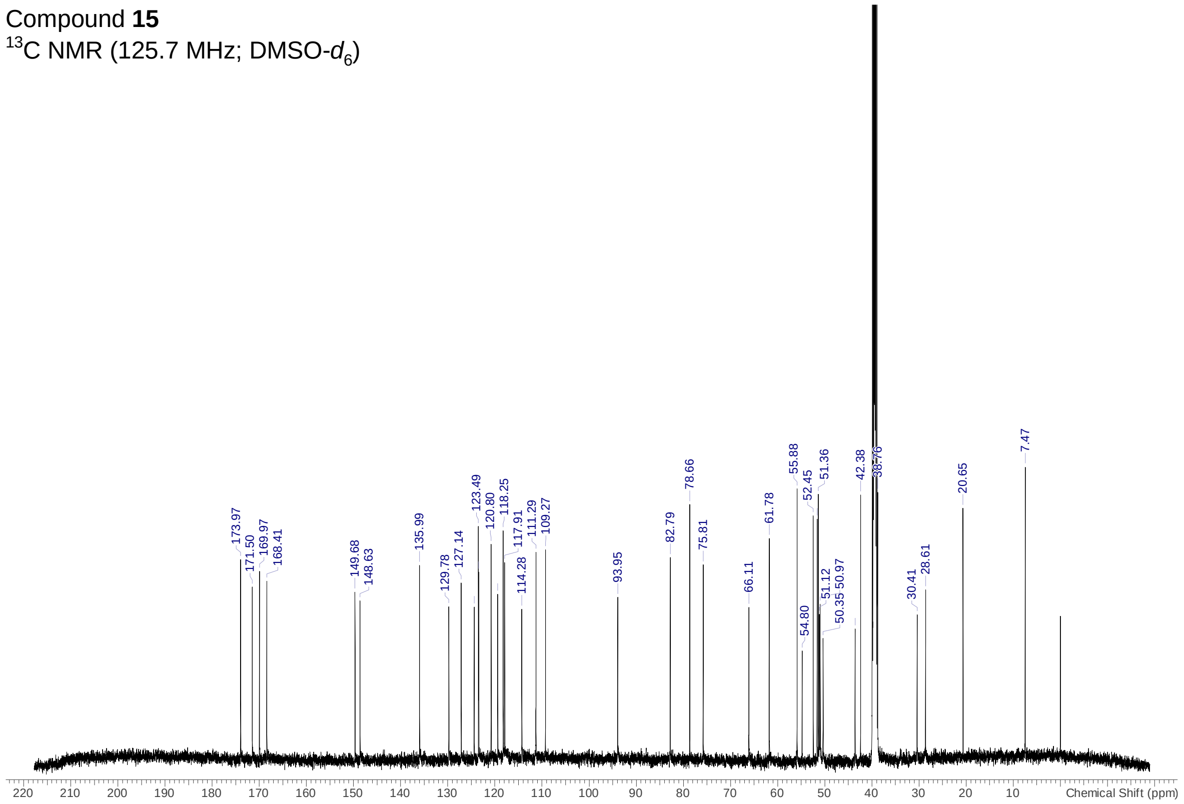

S.9. Compound (17)

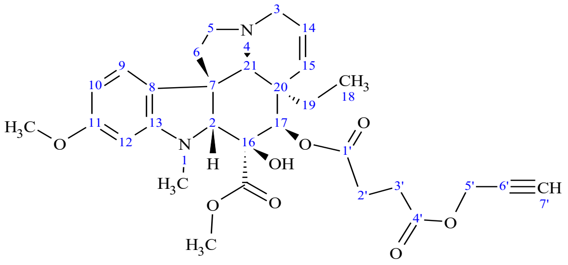

Compound **17**
^{1}H NMR (499.9 MHz; DMSO-d_6)

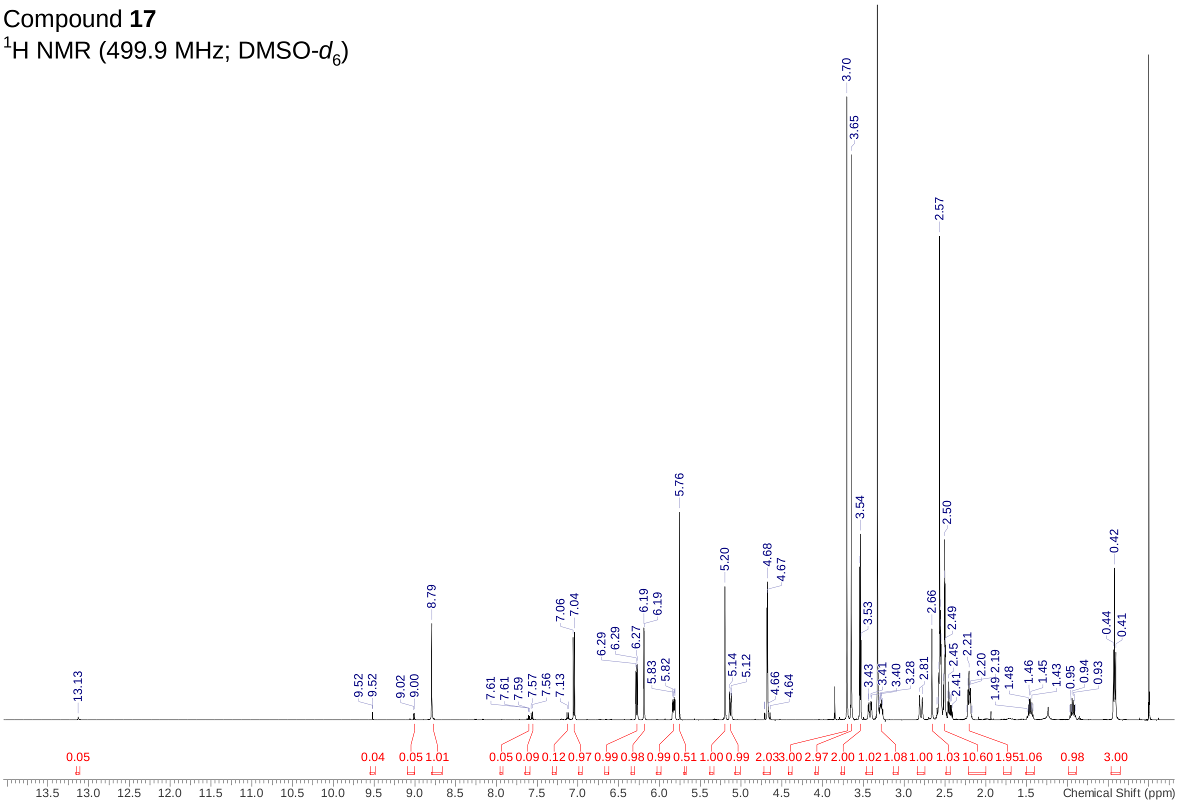

Compound **17**
^{13}C NMR (125.7 MHz; DMSO-d_6)

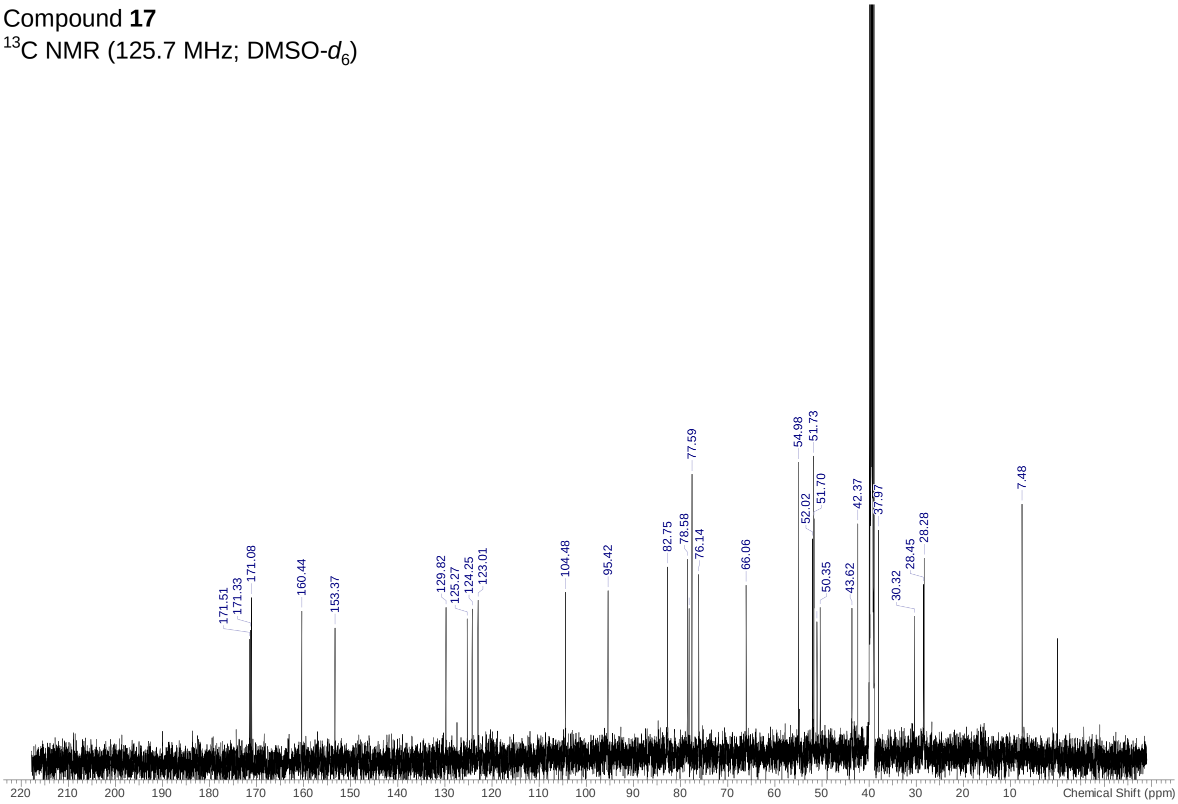

S.10. Compound 19

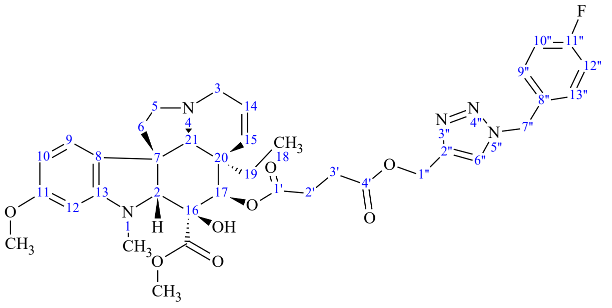

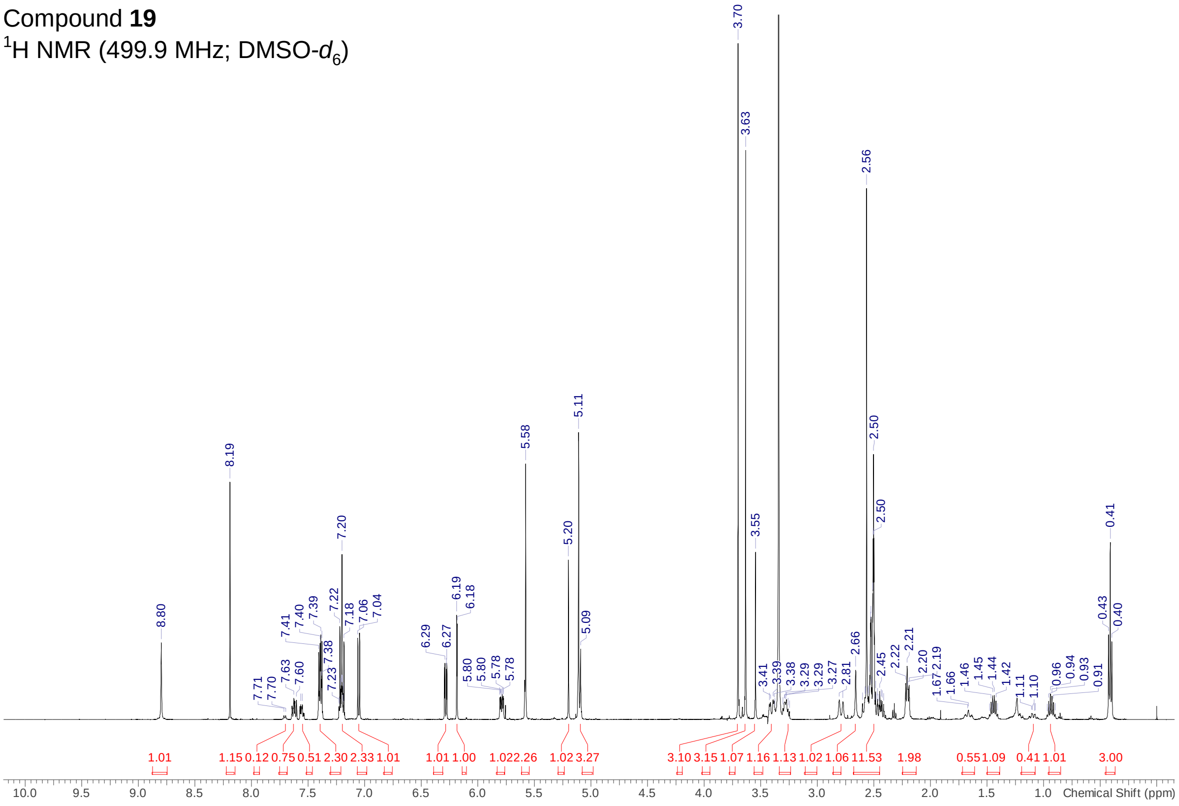

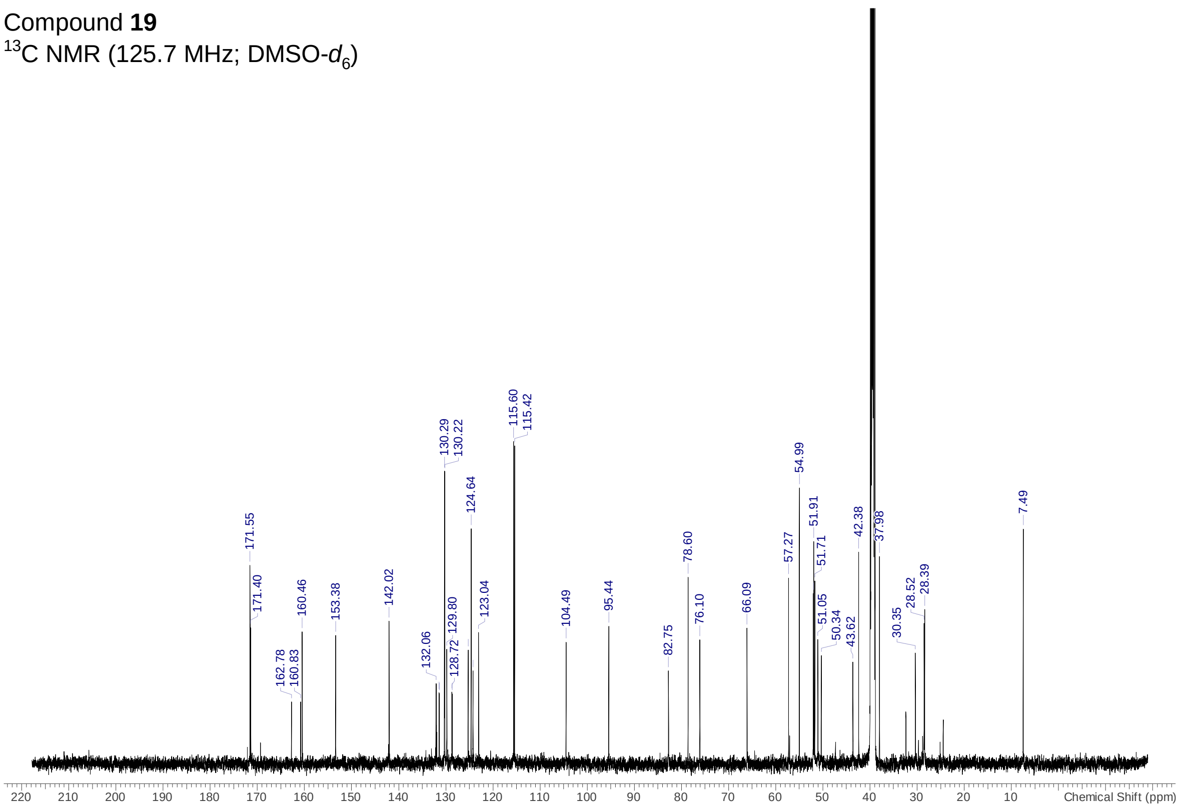

S.11. Compound 20

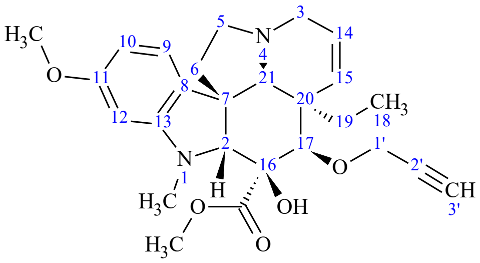

Compound **20**
^{1}H NMR (499.9 MHz; DMSO-d_6)

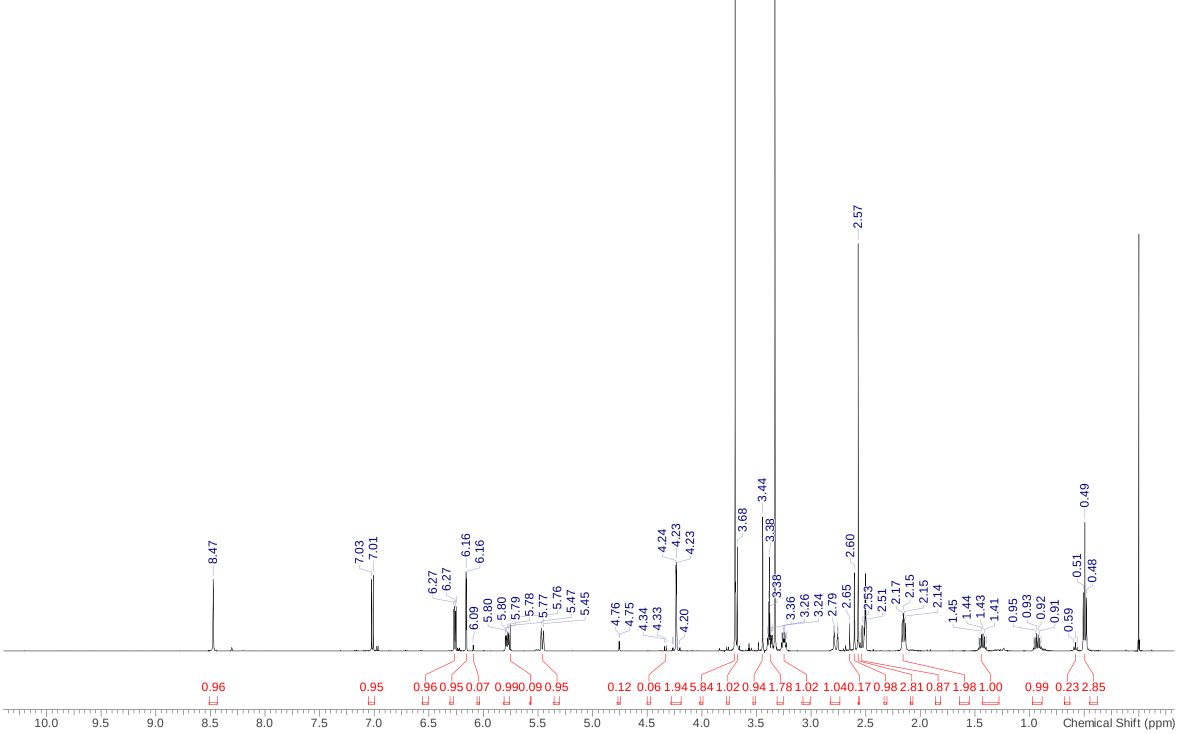

Compound **20**
^{13}C NMR (125.7 MHz; DMSO-d_6)

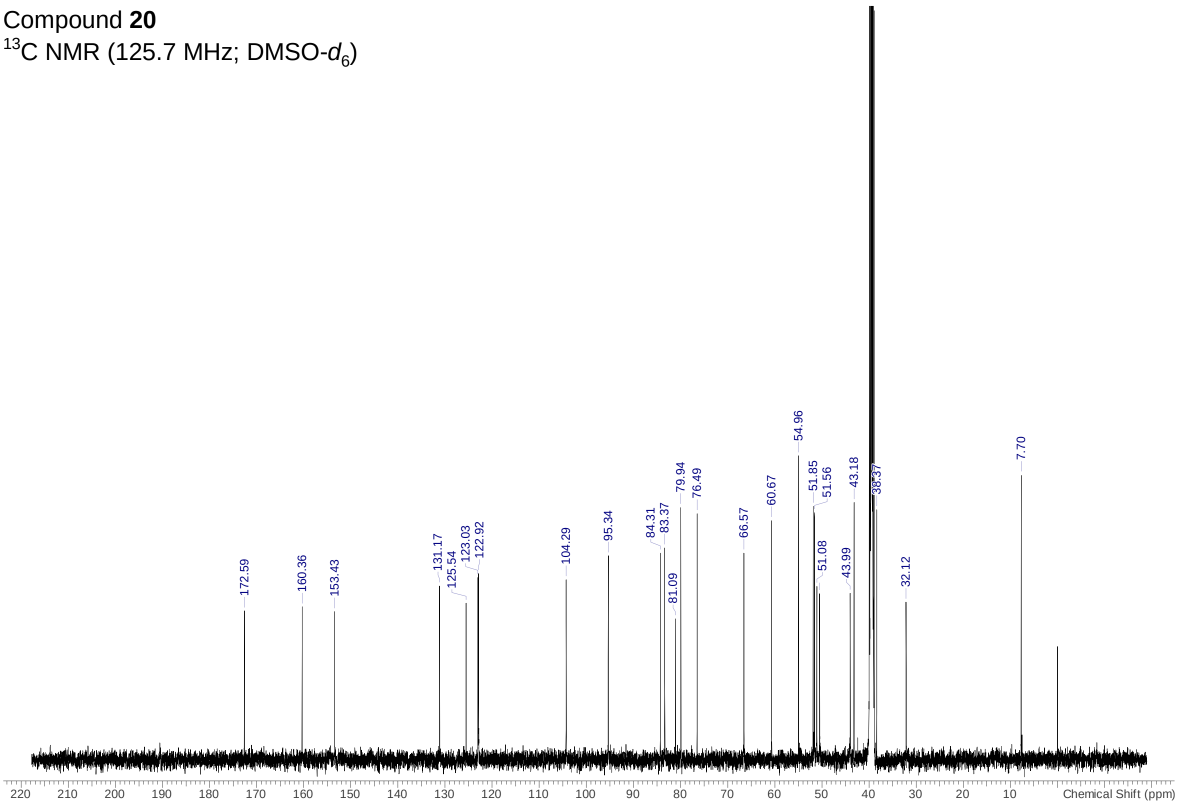

S.12. Compound 21

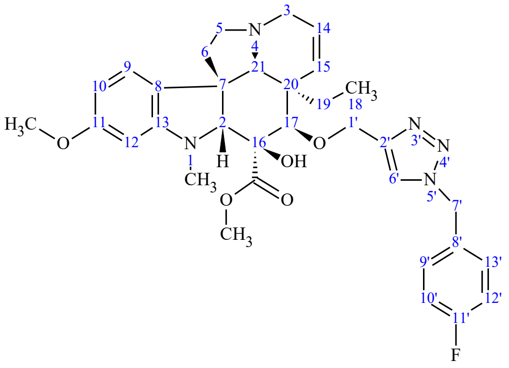

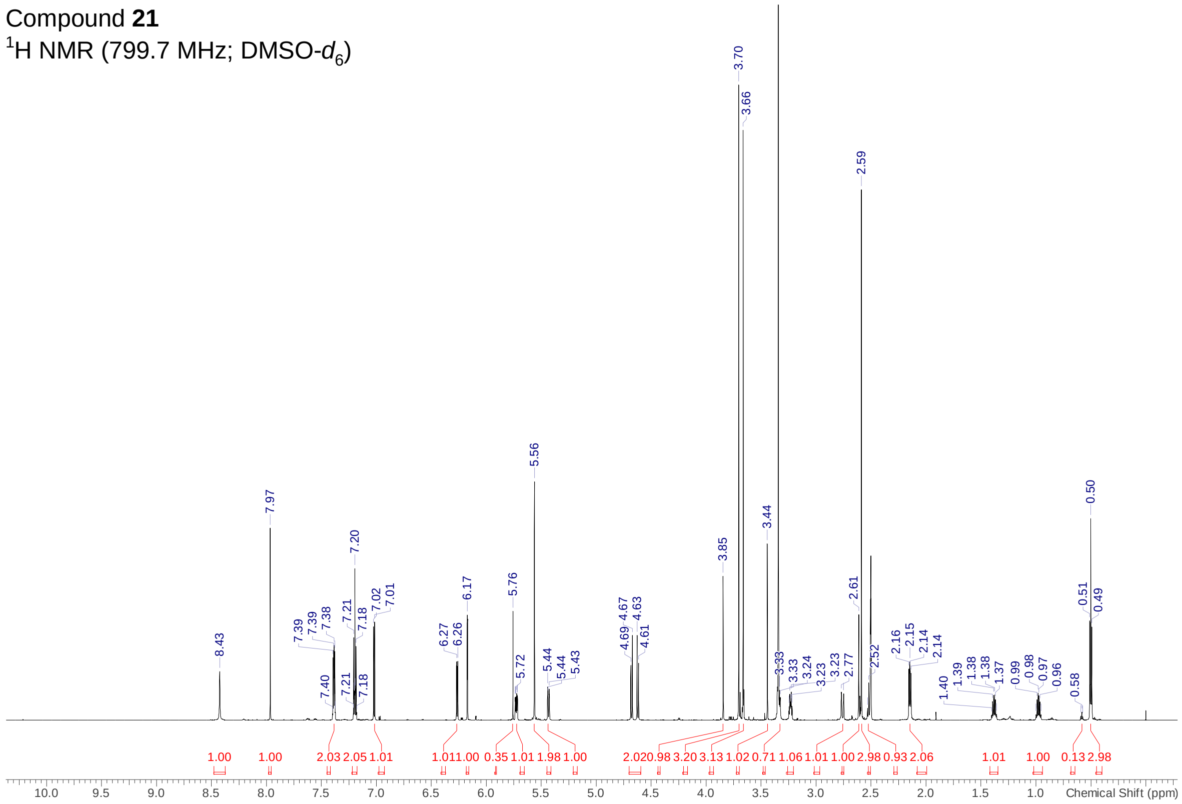

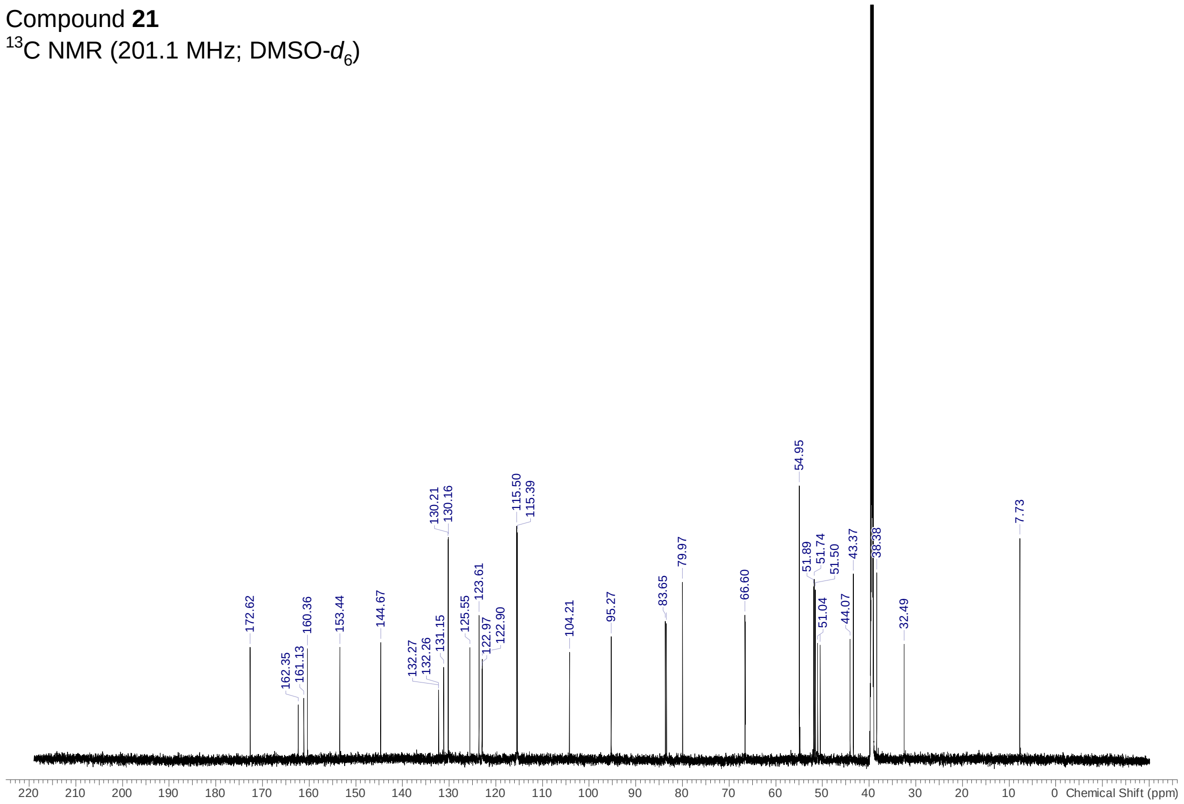

S.13. Compound 23

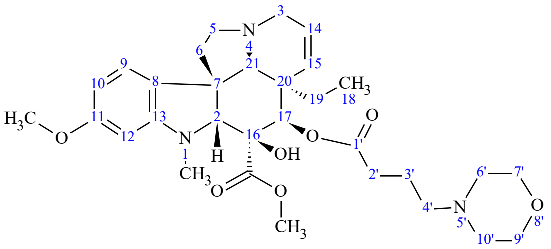

Compound **23**

^1H NMR (499.9 MHz; $CDCl_3$)

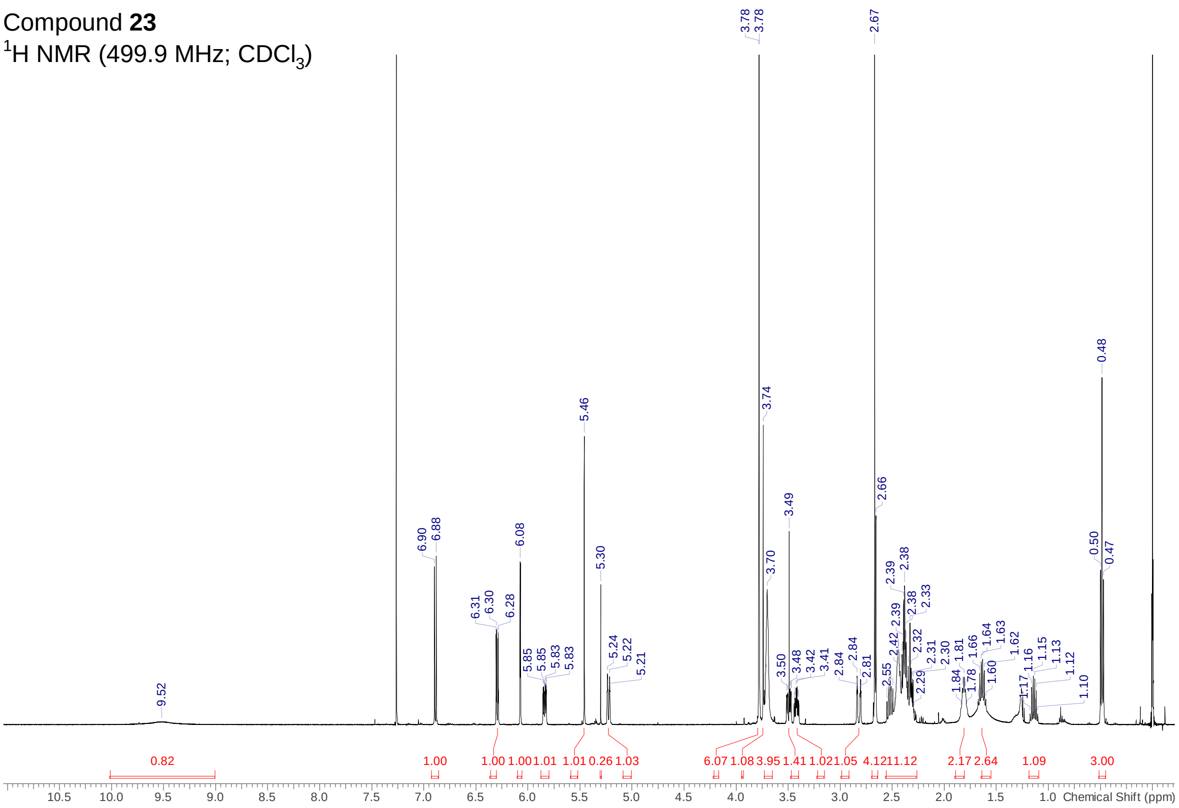

Compound **23**

^{13}C NMR (125.7 MHz; $CDCl_3$)

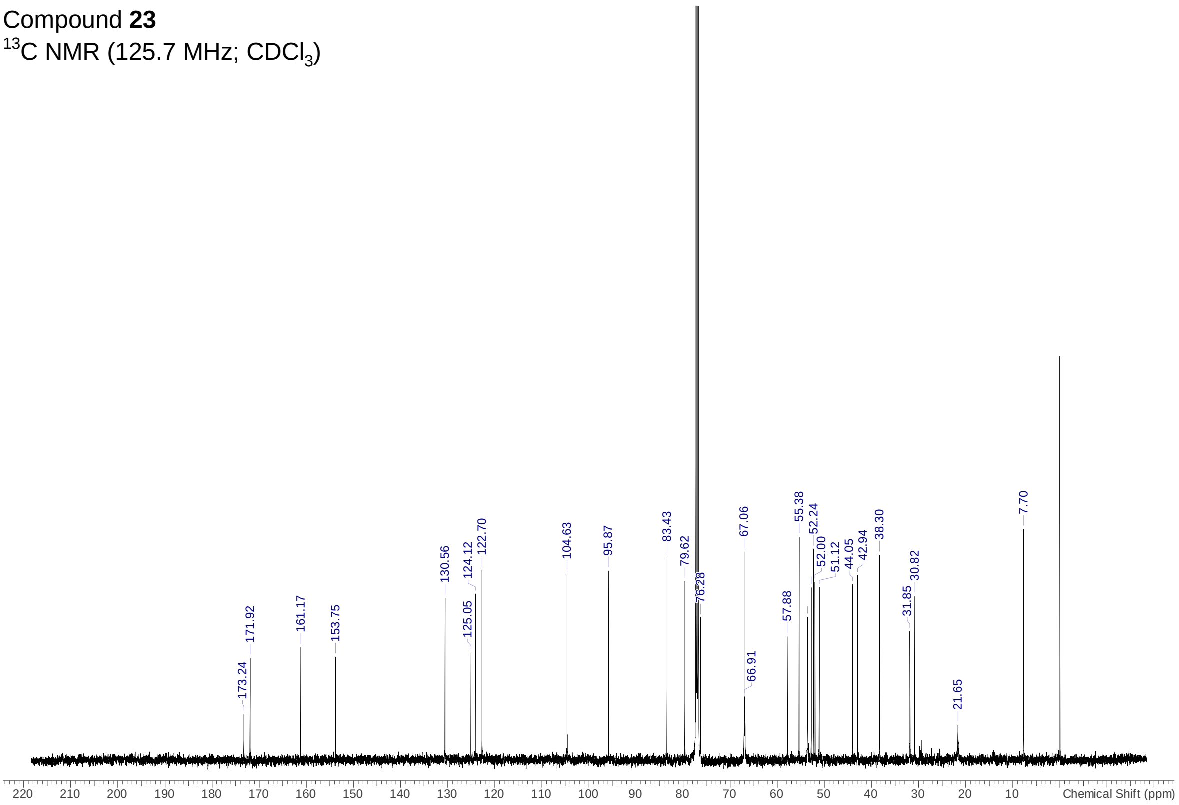

S.14. Compound 24

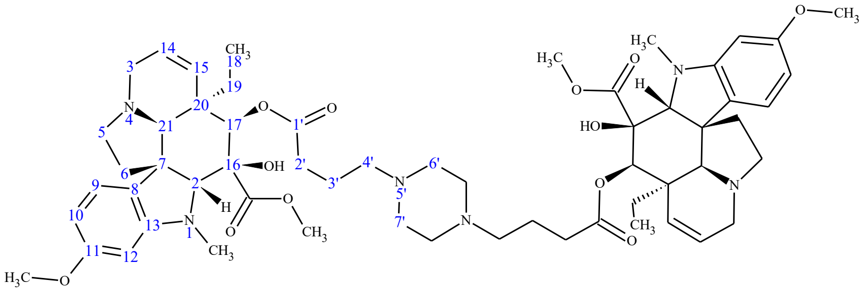

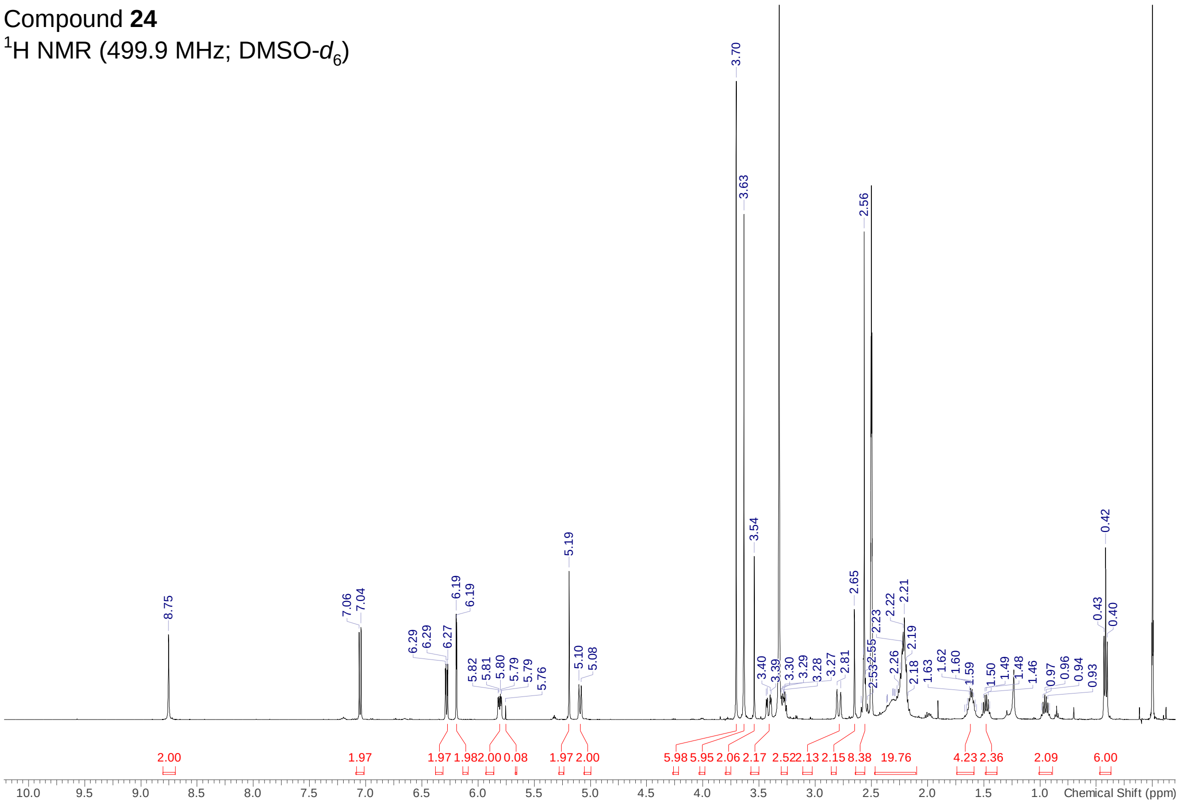

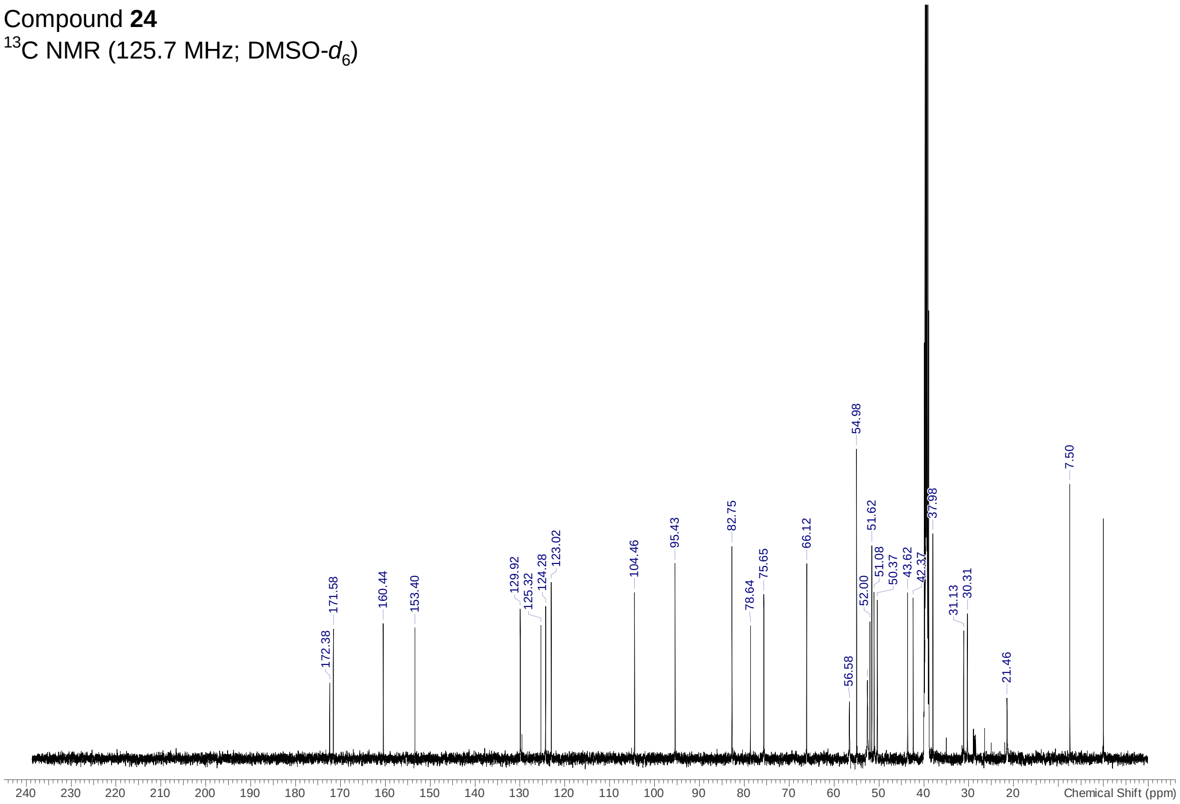

S.15. Compound 25

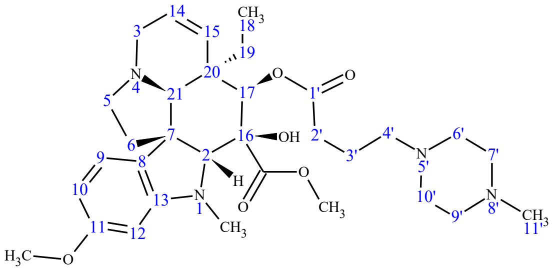

Compound **25**

^{1}H NMR (499.9 MHz; DMSO-d_6)

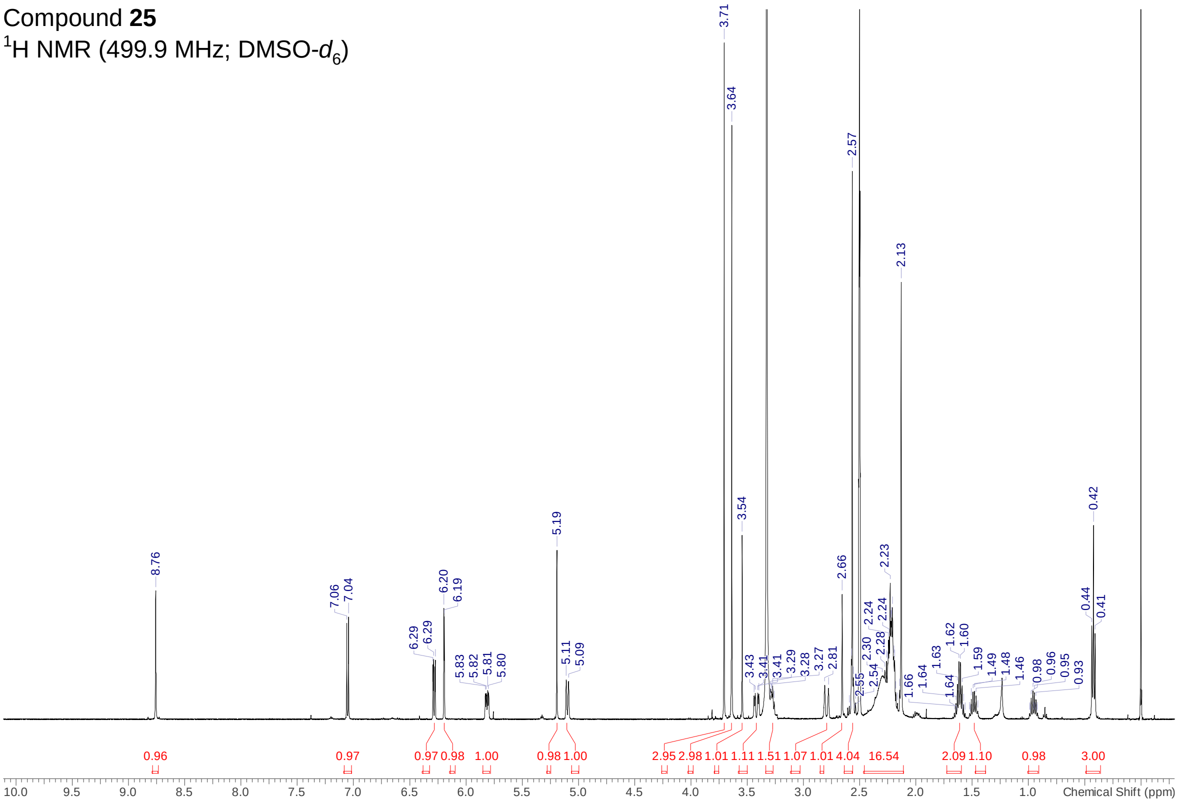

Compound **25**

^{13}C NMR (125.7 MHz; DMSO-d_6)

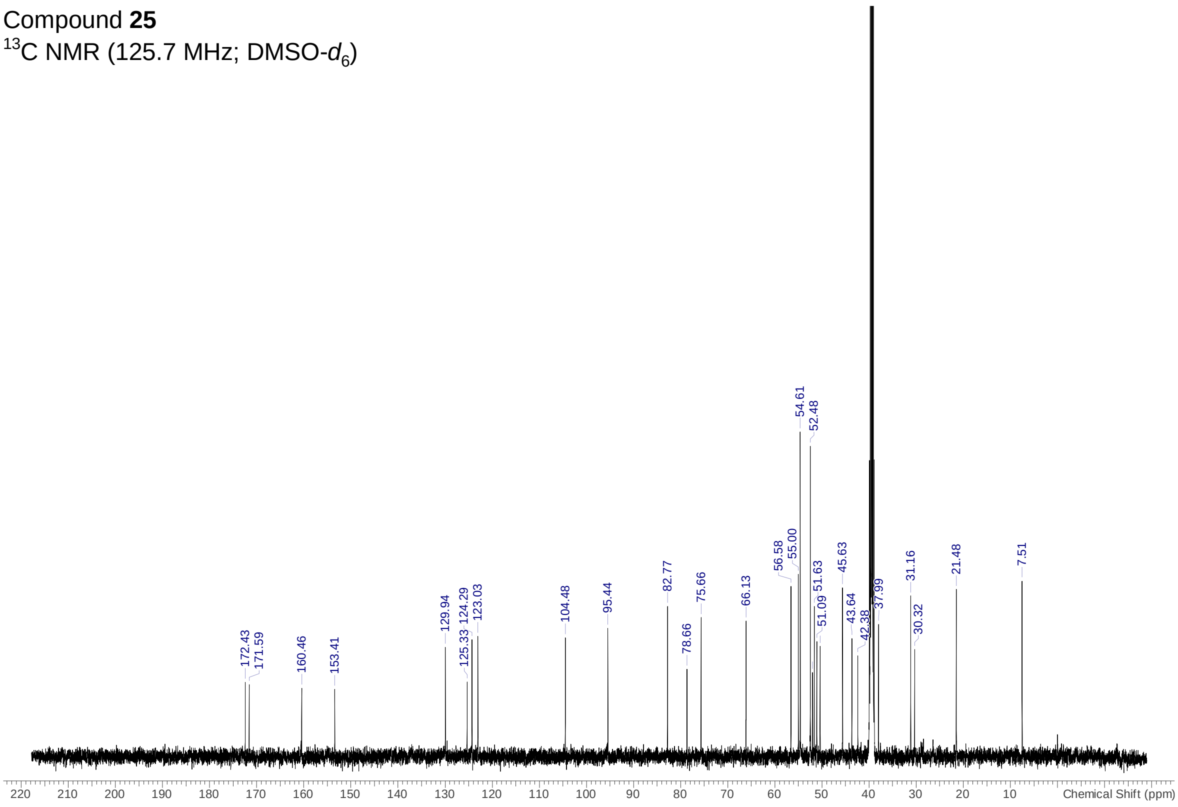

Supplement: Supplementary file 1 [file molecules-25-01010-s001.pdf]
